# Nanomaterials-assisted gene editing and synthetic biology for optimizing the treatment of pulmonary diseases

**DOI:** 10.1186/s12951-024-02627-w

**Published:** 2024-06-18

**Authors:** Lanjie Lei, Wenjie Pan, Xin Shou, Yunyuan Shao, Shuxuan Ye, Junfeng Zhang, Narasaiah Kolliputi, Liyun Shi

**Affiliations:** 1 Key Laboratory of Artificial Organs and Computational Medicine in Zhejiang Province, Institute of Translational Medicine, Zhejiang Shuren University, Hangzhou, Zhejiang 310015 China; 2 Department of Pharmacy, The Third Affiliated Hospital of Wenzhou Medical University, Wenzhou, 325200 China; 3 Department of Immunology and Medical Microbiology, Nanjing University of Chinese Medicine, Nanjing, 210046 China; 4 Division of Allergy and Immunology, Department of Internal Medicine, Morsani College of Medicine, University of South Florida, Tampa, FL 33612 USA

**Keywords:** Nanomaterials, Gene editing, Synthetic biology, Pulmonary diseases

## Abstract

The use of nanomaterials in gene editing and synthetic biology has emerged as a pivotal strategy in the pursuit of refined treatment methodologies for pulmonary disorders. This review discusses the utilization of nanomaterial-assisted gene editing tools and synthetic biology techniques to promote the development of more precise and efficient treatments for pulmonary diseases. First, we briefly outline the characterization of the respiratory system and succinctly describe the principal applications of diverse nanomaterials in lung ailment treatment. Second, we elaborate on gene-editing tools, their configurations, and assorted delivery methods, while delving into the present state of nanomaterial-facilitated gene-editing interventions for a spectrum of pulmonary diseases. Subsequently, we briefly expound on synthetic biology and its deployment in biomedicine, focusing on research advances in the diagnosis and treatment of pulmonary conditions against the backdrop of the coronavirus disease 2019 pandemic. Finally, we summarize the extant lacunae in current research and delineate prospects for advancement in this domain. This holistic approach augments the development of pioneering solutions in lung disease treatment, thereby endowing patients with more efficacious and personalized therapeutic alternatives.

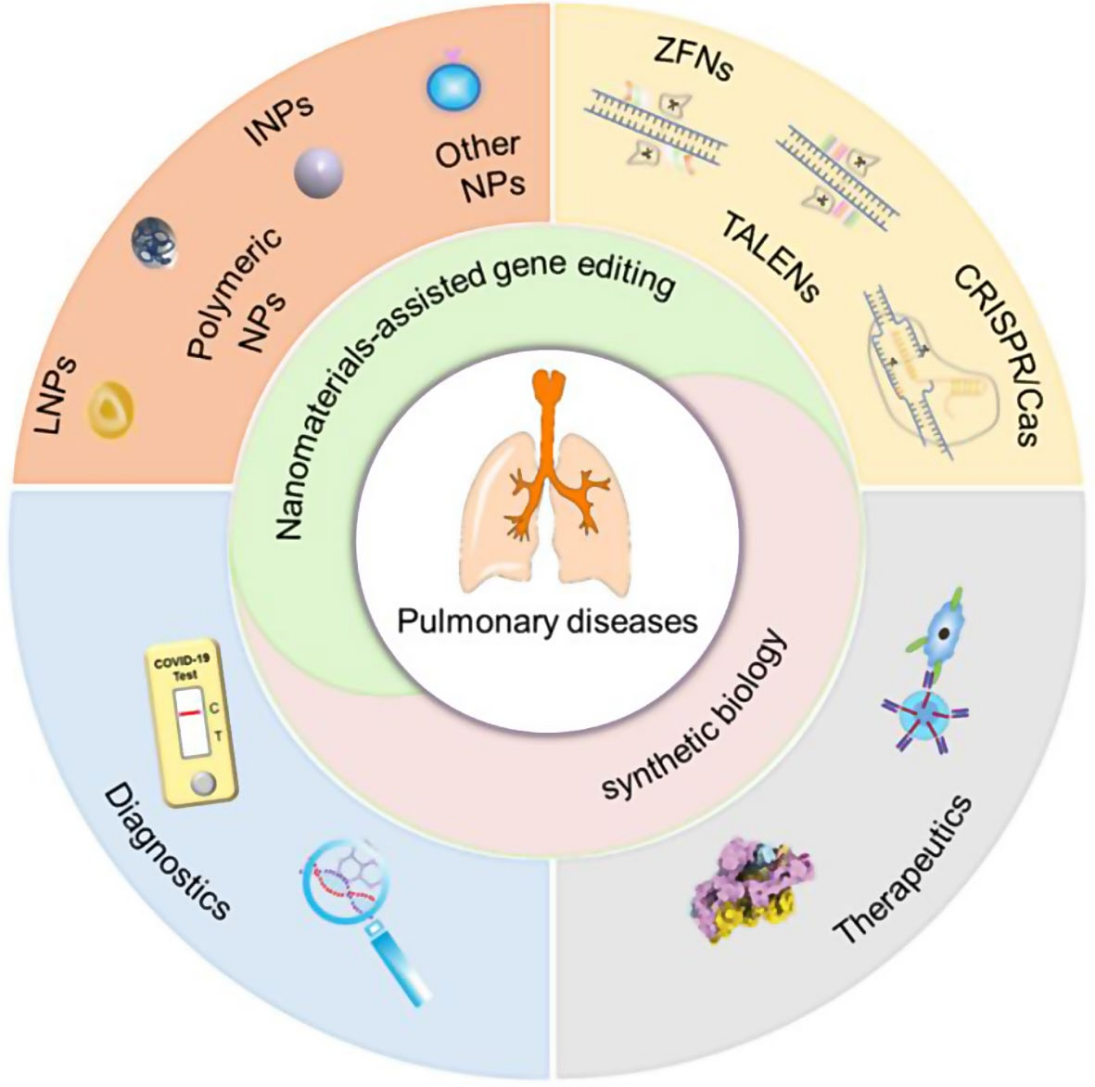

## Introduction

Pulmonary diseases have persistently presented formidable hurdles for public health and healthcare systems [1, 2]. The intricate physiology of the respiratory system renders the targeting of damaged lungs arduous in conventional therapies [3–5]. This underscores the urgent need for innovative strategies to manage lung disease. In this context, drug delivery using nanocarriers is a potentially effective way to avoid conventional therapeutic limitations. Simultaneously, the evolution of advanced biomedical technologies, namely gene editing and synthetic biology, has the potential to augment drug bio-dispersibility and efficacy within target organs, reduce undesired side effects, and unlock novel avenues for enhancing the spectrum of lung disease management [6, 7].

Nanotechnology, an interdisciplinary field spanning physics, chemistry, and biology, offers innovative pathways for addressing biomedical challenges by designing, controlling, and manipulating materials and structures at the nanoscale [8]. In lung disease treatment, nanomaterials are a flexible platform for precise administration of drugs and targeted administration of gene-editing tools, supporting cellular engineering [9–11]. Gene editing has become a focal point in precise genetic modification technology [12]. Notably, the Clustered Regularly Interspaced Short Palindromic Repeats (CRISPR)/ associated protein (Cas)9 system has revolutionized the field of gene editing due to its remarkable efficacy and simplicity. It involves the knockout, insertion, and mutational target genes modification using the Cas9 nucleic acid endonuclease and a single-stranded guide RNA (sgRNA) that targets the gene of interest [13, 14]. With this technology, genome editing is performed with high precision, enabling the study of the relationship between genes and biological traits and treating diseases such as genetic disorders and cancer. Nevertheless, gene-editing tools require precise guidance to effectively modify lung cells, which poses considerable challenges. The emergence of nanomaterials presents a new methodology to address this challenge [15, 16], which involves the utilization of nano-delivery systems to correctly convey gene-editing tools to specific lung cells, consequently reducing unintended effects on the surrounding tissues [17].

Synthetic biology constitutes a design-centric discipline, with its core focus on conceiving novel biological functionalities by discovering, characterizing, and repurposing molecular components [18–20]. In this context, microorganisms can be systematically engineered and tailored through synthetic biological methodologies to enable the production of specific pharmaceutical compounds or serve as active therapeutic agents in the context of lung disease treatment [21–23]. Furthermore, based on the design principles and engineering framework intrinsic to synthetic biology, mammalian cells can be modified to manifest specific functionalities, including those relevant to organ transplantation, cell-based therapies, and vaccine production [24]. Synthetic biology provides broad applications, including vaccine development for lung diseases, molecular diagnostics, and cell-based therapeutic interventions. However, synthetic biology faces several technical and ethical challenges as a cutting-edge technology in life sciences while bringing benefits and visions to human society. Synthetic biology requires a very high level of understanding of biological fundamentals. Scientists need a comprehensive understanding of a biological system’s structure and function to accurately design and construct new biological systems [25]. At the same time, synthetic biology is concerned with the nature of life and the design of living organisms, raising discussions about bioethics and biodiversity conservation. In addition, the accidental release or deliberate misuse of synthetic organisms may lead to unknown biosafety risks [26]. Therefore, while encouraging its development, there is a need to strengthen relevant safety regulations and ethical constraints so that the potential of synthetic biology can be maximized while reducing the risks it poses.

Here, we highlight the potential of nanomaterials to enhance gene editing and synthetic biology, as well as present innovative approaches for lung disease treatment. Firstly, an overview of the respiratory system and limitations of present treatments for pulmonary disorders are briefly described. The application of nano-delivery systems in pulmonary diseases is also reviewed, along with insights into the rationale underlying the nano-delivery systems design for pulmonary therapy. Secondly, we outline the application of nanomaterial-assisted gene editing and synthetic biology in developing novel therapies for pulmonary diseases. Finally, we propose the prospects for utilizing these techniques for lung disease treatment and their possible clinical applications.

## Respiratory system overview

The efficacy of pulmonary therapy is hindered by respiratory physiology [27]. The upper respiratory system, which encompasses the nose, mouth, pharynx, and larynx, regulates lung-bound airflow and filters the incoming air [28]. The lower respiratory tract, which comprises the trachea and lungs, is a complicated network of branching airways characterized by a bronchial tree interlinked with the alveolar system. This intricate structure presents a multitude of barriers that shield the lungs from potential environmental hazards [29], particularly relevant are the airways host mucus and cilia, which constitute a physical barrier that intercepts particulate matter and microorganisms from lung entry [30]. While mucus traps irritants or inhaled substances, the coordinated motion of cilia propels mucus out of the lungs, maintaining lung hygiene [31]. Meanwhile, in obstructive lung diseases, the thickening of the mucus layer due to bronchoconstriction and increased mucus secretion disrupts the drug delivery mechanism [32].

When the delivered drug avoids mucosal ciliary clearance, it effectively engages with the target tissue; however, the intricate pulmonary environment, replete with various compounds, such as surfactants and protein hydrolases, presents obstacles, limits drug adsorption, and triggers its deactivation [33]. Alveolar surfactants comprise lipids and proteins that interact with drugs or particles [34]. Consequently, they can encase the drug with protein crowns, thwart adsorption, and expedite its elimination by alveolar macrophages. Furthermore, the pulmonary arena hosts an array of immune cells and molecules, including macrophages, lymphocytes, and immunoglobulins. These entities can phagocytose and degrade biological macromolecules and particles [35]. Notably, heightened macrophage activity is crucial in the pathogenesis of specific pulmonary diseases such as infection, acute lung injury, and chronic obstructive pulmonary disease (COPD) [36]. In summary, insights into the characterization of the lung barrier could promote the development of more effective lung therapies. Nanomaterial-based therapy is a promising alternative to traditional treatments with limited effectiveness in delivering drugs to the lungs [37, 38]. Nanomaterials have nanoscale dimensions and move more freely in the body. In addition, nano-delivery systems can enhance the stability of therapeutic agents, including DNA and RNA, to protect against early degradation and rapid clearance in vivo [39]. This allows the drug formulation to be delivered to the target region, enabling precise drug control, reducing toxic side effects, controlling biodistribution, and accelerating drug action or response. Several nanomaterials are available to deliver gene editing tools, offering potential solutions for treating lung diseases.

## Nano-delivery system for pulmonary diseases treatment

### Nano-delivery system

Nanotechnology in medicine offers new treatment strategies for lung disease [40]. Nanomedicine refers to the use of nanotechnology in healthcare and related research for supervising, regulating, constructing, repairing, protecting, and enhancing biological systems at the molecular level [41]. Nanotechnology has many unique physicochemical properties due to the quantum effects of materials at the nanoscale, which provide unlimited possibilities for the preparation, performance, improvement, and application of nanoscale products [42]. Within this framework, nano-delivery systems present a viable means of transporting drugs or messenger ribonucleic acid (mRNA) to a specific target [43, 44]. Nanomaterials can function as carriers to facilitate the delivery of diagnostic agents.

Nanomaterials offer a compelling avenue for developing controlled-release delivery systems suitable for the pulmonary environment [45]. Their small scale of nanomaterials imparts novel functionalities to nanomedicine [46]. Specifically, nanocarriers enhance the stability of active agents during transport, safeguarding them from extracellular enzymes and evading scavenging systems [47]. These carriers facilitate the cellular uptake of active compounds and enable their controlled and targeted delivery with uniform distribution, thereby extending the retention time within the target tissue and mitigating adverse effects through protective shielding [48]. Collectively, these attributes synergistically enhance the pharmacokinetics and pharmacodynamics of active compounds. Furthermore, a diverse array of nanomaterials is already being used to overcome the limitations of lung therapies. The substantial absorption, extensive circulation, and permeability of the lungs promote nanoparticles (NPs) accumulation in the airways and lungs [49]. These unique characteristics make nanomaterials potential tools for lung therapy.

### Types of nano-delivery systems

**Fig. 1:**
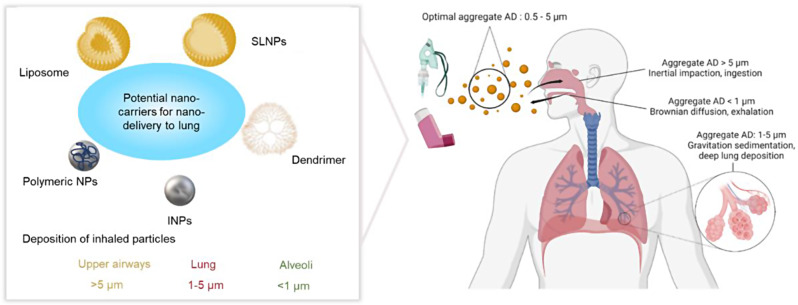
Schematic representation of potential nanocarriers and their AD-dependent deposition and distribution mechanisms in the respiratory system. Adapted from Refs [42, 50]

With the rapid advancements in biomaterials, nanocarriers designed for enhanced delivery, including lipids [50–52], polymers [53], inorganic NPs (INPs) [54], and other nanomaterials [55], have demonstrated substantial potential. The nanocarrier size is an key parameter that affects its deposition in the lungs (Fig. 1). The aerodynamic diameter (AD) represents the size of the atomized particles, which determines the region of the respiratory system where particle deposition occurs. Additionally, selecting delivery carrier strategies must consider three crucial aspects: biosafety, delivery efficiency, and target specificity [56]. To ensure biosafety, it is imperative to select carriers that exhibit biocompatibility and minimal immunogenicity [57]. Furthermore, the chosen carrier must be able to facilitate targeted therapy within the lungs while surmounting inherent biological and physical pulmonary barriers [58]. These include enhancing drug solubility and extending the duration of drug retention, thereby optimizing therapeutic outcomes.

#### Lipid-based nanomaterials

Lipid-based nanomaterials are nanoparticle carriers prepared using biodegradable synthetic lipid molecules that can carry, deliver, and release hydrophobic and lipophilic preparations [59]. Depending on their nanostructure and lipid source, these materials can be categorized into liposomes, lipid NPs (LNPs), and other carriers. Initially discovered by Bangham in 1965, liposomes exhibit biocompatibility and low toxicity as their composition resembles cell membranes and lung surfactants. Their lipid-like nature facilitates the traversal of biological barriers and enhances absorption [60, 61].

Liposomes come in different forms. Multilamellar vesicles have multiple lipid bilayers and are 500–5,000 nm in size [62]. Single lipid bilayer unilamellar vesicles ranging in size from 100 to 800 nm [63]. Long-circulating liposomes are strategically engineered with surface polymers to bolster their circulatory stability, whereas immunoliposomes equipped with antibody coatings attain precision in targeting specific cell types. The key advantages of liposomes over conventional therapies include drug protection against degradation, precise drug and macromolecule targeting, and reduced drug cytotoxicity [59, 64]. Notably, liposomes serve as prominent carriers in cancer therapy, and Doxil® was the first polyethylene glycol (PEG)-modified long-circulating liposome approved by the Food and Drug Administration (FDA) for cancer therapy in 1995 [42, 43, 65–67]. Liposomes encapsulate diverse drugs, including antibiotics, bronchodilators, immunosuppressants, anticancer agents, sex hormones, peptides, proteins, and oligonucleotides [68]. Despite their established application in clinical drug delivery, liposomes exhibit reduced delivery efficiency compared with viral vectors [69]. This phenomenon arises mainly from the multifaceted challenges that liposomes encounter upon interacting with cellular membranes, including factors such as membrane mass, charge, and liposome stability. Furthermore, efficient drug release from the liposomal interior is a potential compromise since the drug must traverse the lipid bilayer to access the cell. Researchers have consistently explored novel methodologies and techniques to augment the efficiency of liposomal delivery. Notably, enhancing liposome composition, dimensions, and surface characteristics enhances cellular interactions. Moreover, optimizing liposomal drug delivery efficacy can be further enhanced through strategic amalgamation with other modalities, such as targeted ligands and facilitated delivery systems.

Advances in nanotechnology have spurred the transformation of liposomes into versatile LNPs. Cationic liposomes are positively charged and can adsorb negatively charged nucleic acids or proteins through electrostatic interaction [70, 71]. Cellular internalization is expedited by endocytosis. This promotes the role of liposomes in the delivery of drugs, genes, and various biomolecules. Unlike viral vectors, cationic lipid liposomes offer distinct advantages such as mitigated off-target effects, reduced immunogenicity, enhanced biocompatibility, and elevated cargo capacity [72, 73]. Nevertheless, an overabundance of cationic lipids or imbalanced cation-nucleic acid ratios warrants attention, as these can precipitate cytotoxicity. Additionally, serum proteins may affect transfection efficiency, while the cationic lipid-deoxyribonucleic acid (DNA) complexes stability is diminished [74].

The emergence of ionizable lipids was a pivotal advancement in the developing LNPs [75]. The polarity of ionizable liposomes undergoes pH-dependent alterations. Under acidic conditions, ionizable liposomes adopt a positive charge that enables the formation of mRNA complexes that serve as mRNA stabilizers but assume a neutral charge at physiological pH, which mitigates their potential toxicity [76]. Furthermore, the fusion of zwitterionic liposomes possessing low apparent charges with cationic liposomes can bolster the stabilization of the nanocarriers within the extra-cellular environment and heighten the load efficiency [77]. Miller et al. innovatively synthesized amphoteric amino lipids [78]. These novel compounds comprise amphoteric sulfobetaine head groups, amine-rich linker regions, and diverse hydrophobic tails that collectively enhance the efficacy of nucleic acid delivery. The resulting vectors demonstrated remarkable potential for gene expression using mRNA, showing robust outcomes both in vitro and in vivo.

Solid lipid NPs (SLNs) are usually based on natural or synthetic lipids such as lecithin and triacylglycerol, which are characterized by low toxicity, biocompatibility, and biodegradability [79]. Unlike the liposomal bilayer structure, in which phospholipids are the main component, SLNs are solid particles formed from various lipid-like materials, which can encapsulate hydrophobic drugs and improve drug bioavailability. Since the active ingredient is encapsulated in the solid lipid matrix, drug release from SLNs is controlled by the physicochemical properties of the lipid matrix, such as its crystallinity, particle size, and surface area. Therefore, SLNs are often used as intravenous or topical administration carriers to achieve targeting and controlled release [80]. Furthermore, the emulsion layer around the SLNs enhances the interaction of the NPs with the target sites and improves the selectivity of drug delivery. Specific ligands modify the surface of SLNs, which enhances the SLNs interaction with the target cells [81]. Therefore, SLNs are potential carriers for systemic or localized delivery of therapeutic agents [82].

**Fig. 2:**
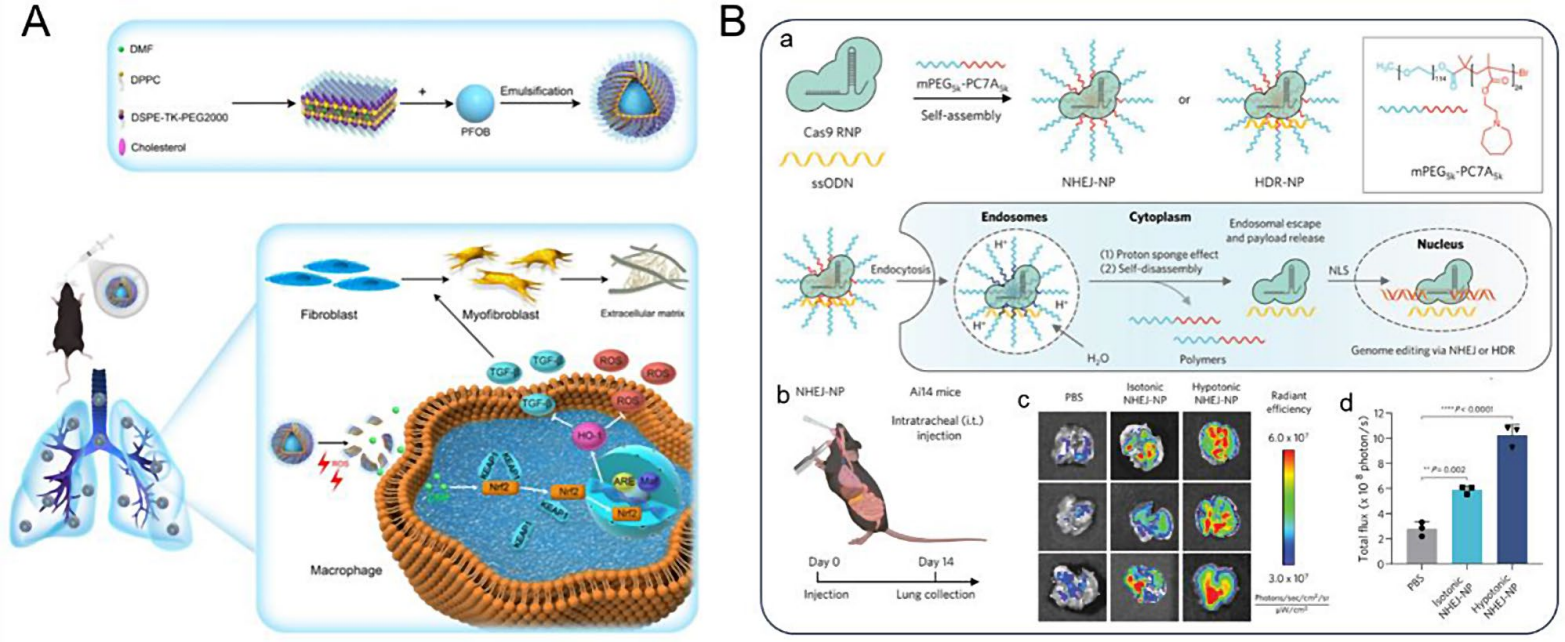
(**A**) Schematic representation of ROS responsive liposome-DTP@DMF NPs synthesis and therapeutic mechanism [83]. (**B**) pH-responsive polymer delivery system methoxy-PEG (mPEG)-pH-sensitive polymer bearing a seven-membered ring with a tertiary amine (PC7A) NPs for Cas9 ribonucleoprotein (RNP) and single-strand oligonucleotides (ssODN) delivery [86]. (a) Schematic representation of mPEG-PC7A NPs synthesis and entry into cells. (b-d) Gene editing in vivo using delivering Cas9 RNP alone (non-homologous end joining-NP) in Ai14 mouse lungs by intratracheal injection. Reprinted with permission from Ref [83, 86]

In conclusion, recent advances in liposomal technology have resulted in significant breakthroughs, primarily driven by the development of diverse arrays of LNPs. This progress has been further augmented by the emergence of stimuli-responsive liposomes, a novel class of carriers that facilitate efficient drug delivery in lung diseases [83, 84]. For example, Wang et al. designed reactive oxygen species (ROS)-responsive 1,2-distearoyl-sn-glycero-3-phosphoethanolamine (DSPE)- thymidine kinase (TK)-PEG@ dimethyl fumarate (DMF) liposomes (DTP@DMF NPs) containing the nuclear factor erythroid 2-related factor 2 (Nrf2) agonist DMF (Fig. 2A) [83]. It was demonstrated that this ROS-responsive liposome is an ideal inhaled drug delivery system for effectively treating pulmonary fibrosis. Furthermore, the strategic integration with liposomes and complementary biomaterials is one of the most promising strategies for designing more efficient drug carriers [85].

#### Polymeric NPs

Polymer NPs are solid micelles composed of natural, synthetic, or semi-synthetic biodegradable polymers [87]. These NPs possess diverse chemical and physical attributes that can be customized for specific applications [88]. Achieving an optimal polymer-based delivery system requires meticulous consideration of physicochemical aspects such as polymer system shape, porosity, size distribution, surface morphology, crystallinity, surface charge, copolymer component, and coating material nature [89]. Polymer NPs are usually formed by amphiphilic self-assembly inclusion molecules with hydrophobic polymers through hydrophobic-hydrophilic interactions. Drug encapsulation using polymer NPs typically occurs in the form of nanocapsules, nanospheres, or polymeric micelles [90]. Nanospheres are characterized by a solid spherical morphology and can bind molecules either on their surfaces or within themselves. In contrast, nanocapsules comprise a solid shell enclosing a liquid core and offer versatility in encapsulating drugs, biomolecules, and other agents [91]. The shell predominantly consists of lipids, proteins, or polymers, thereby safeguarding the encapsulated core. Polymer micelles form due to the uneven distribution of hydrophilic and hydrophobic properties of the polymer molecules. The hydrophobic segments of the polymer chains aggregate to form the core, whereas the hydrophilic segments are oriented towards the aqueous phase. This distinctive arrangement enables effective transportation of hydrophobic agents, including drugs, within the aqueous phase [92].

Polymers are categorized into natural and synthetic [93]. Natural polymers (such as chitosan, sodium alginate, and gelatin) are preferred owing to their superior cytocompatibility and biodegradability. It has been shown that chitosan and alginate enhance nebulization and simplify preparation for pulmonary delivery [94–96]. However, natural polymers exhibit certain limitations. Notably, lung exposure to chitosan and its derivatives has raised safety concerns, whereas alginate administration entails rapid release, which is undesirable for managing persistent infections. In contrast, synthetic polymers offer an alternative to natural materials, affording enhanced control over the delivery profiles and release kinetics [97]. This empowers synthetic polymers to achieve more efficient and sustained drug release over time. In addition, polymer NPs are also used for gene delivery. Xie et al. developed a pH-responsive amphiphilic polymer, methoxy-PEG (mPEG)-pH-sensitive polymer bearing a seven-membered ring with a tertiary amine (PC7A) NPs (Fig. 2B), that can adapt to the heterogeneity of Cas9 RNP and single-strand oligonucleotides (ssODN) through electrostatic and hydrophobic interactions [86]. Cells can uptake the NPs through an endocytosis process and can deliver genome editors to the lungs for cystic fibrosis gene therapy.

Polymers are pivotal part in pulmonary drug delivery with advantages such as manipulable surface properties, protection of drugs from degradation, prolonging drug efficacy, and facilitating sustained drug delivery [98, 99]. For instance, leveraging the homogeneity of the three-dimensional structure of dendrimer macromolecules allows for the incorporation of diverse bioactive agents, yielding bioactive conjugates [100]. This affords dendrimer macromolecules an efficient conveyance of drugs with varying solubilities. Moreover, these polymers can treat the inflammatory respiratory conditions associated with asthma [101, 102]. Additionally, these have been extensively explored as carriers of pulmonary medications, including anti-asthma, anti-tuberculosis, pulmonary arterial hypertension, and anti-cancer drugs. Furthermore, the FDA has authorized the use of poly(lactic-co-glycolic acid) (PLGA) for gene transfer, endorsing its efficacy as a non-viral carrier [103, 104]. Notwithstanding these accomplishments, challenges persist for various polymeric materials, including the dose-dependent toxicity of dendritic macromolecules and mechanical deficiencies of natural polymers.

#### Inorganic NPs (INPs)

INPs are synthesized from inorganic particles and biodegradable polycations [105], including metals, metal oxides, carbon materials, and magnetic nanoparticles such as superparamagnetic iron oxide NPs [106]. Their inherent biocompatibility, stability, resistance to microbial degradation, and efficient delivery contribute to their clinical significance [107]. INPs encapsulate drugs or biomolecules that are subsequently delivered and released through endocytosis across cell membranes, thereby enabling disease treatment. Furthermore, INPs are gene carriers that encapsulate, concentrate, and protect nucleic acids from nuclease degradation [108]. Surface modification not only enhances transfection efficiency but also substantially mitigates NP toxicity. In recent years, gold nanoparticles (AuNPs) [109], mesoporous silica nanoparticles (MSNs) [36], and metal-organic frameworks (MOFs) [110] have gained great attention as nano-delivery systems for respiratory disease therapy. This preference arises from their notable biocompatibility and high specific surface area.

**Fig. 3:**
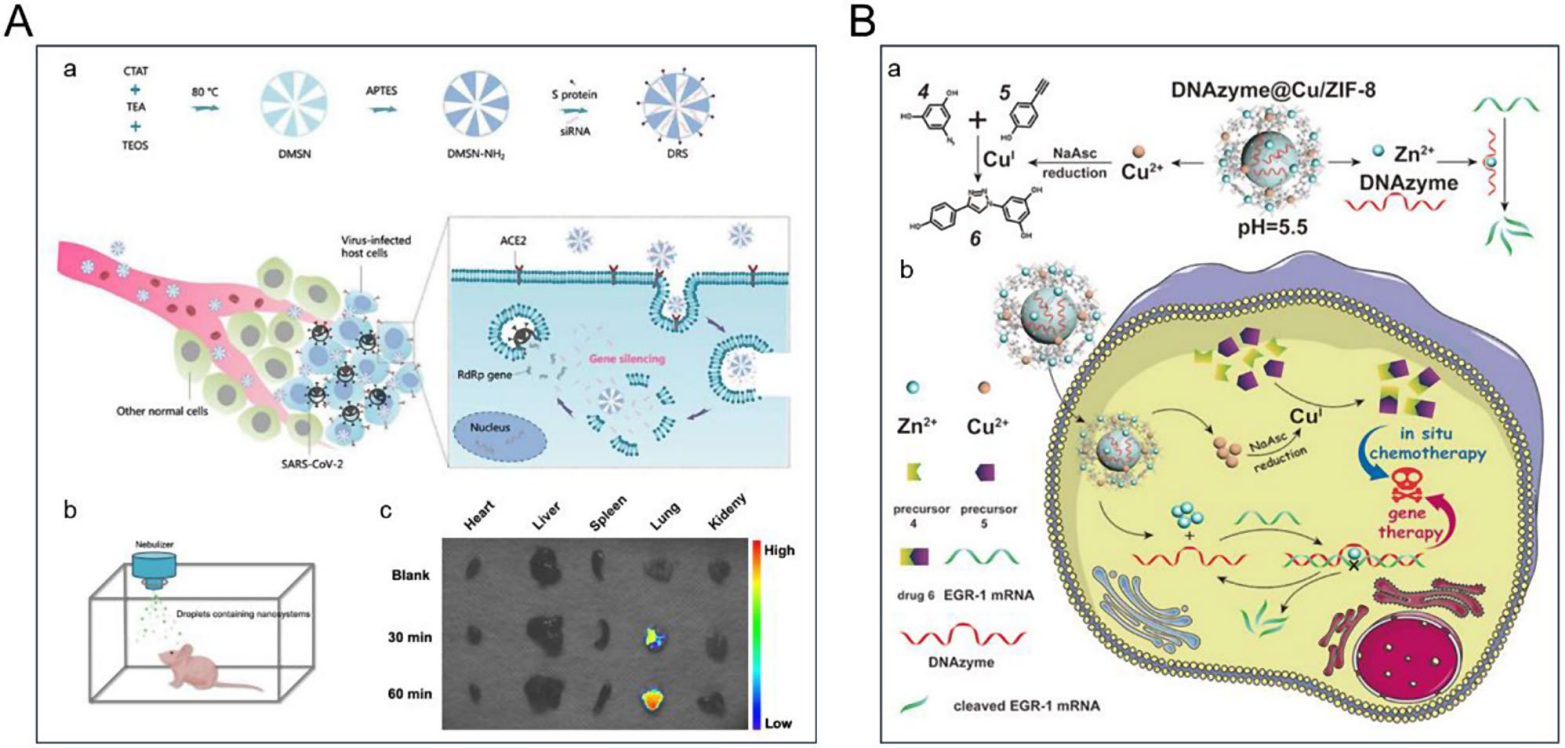
(**A**) Coronavirus S protein-modified dendritic MSNs to deliver specific siRNAs to treat SARS-CoV-2 infection [114]. (**B**) Cu/Zn bimetallic MOF nanoplatforms capable of encapsulating therapeutic deoxyribozymes (DNAzymes) for intracellular drug synthesis and gene therapy [116]. Reprinted with permission from Ref [114, 116]

AuNPs present a range of benefits, including adjustable size, chemical stability, excellent biocompatibility, and extensive surface area, rendering them widely acclaimed as efficacious platforms for drug delivery [109]. Notably, AuNPs are considered relatively non-toxic compared with lipids and polymer NPs [111]. Their appealing attributes include size adjustability and efficient delivery of biomolecules [112]. MSNs possess a structured porosity. These attributes include stability, robust biocompatibility, expansive surface area, and substantial loading capacity. In addition, MSNs biodegrade in vivo, and their exterior surfaces are easily functionalized [113]. In the domain of lung delivery applications, silica-based nanomaterials naturally accumulate within the pulmonary tissue owing to heightened organ vascularity, permeability, and retention capabilities. This inherent property makes them suitable nanocarriers for lung disease treatment [36]. For instance, Zhu et al. prepared a dendritic MSNs modified with coronavirus spiny S protein for effective and targeted delivery of specific small interfering RNAs (siRNAs), which achieved efficient silencing of severe acute respiratory syndrome coronavirus 2 (SARS-CoV-2) related genes **(**Fig. 3A**)**. It provides a new reference for the clinical treatment of SARS-CoV-2 infection [114]. MSNs effectively transport rifampicin, an antibiotic, into the pulmonary system [115]. However, despite the significant benefits arising from the precise control over particle attributes, noteworthy drawbacks are inherent to this approach. Specific synthetic processes employ harsh reagents and solvents, thereby raising concerns regarding residual toxicity. Moreover, the potential attenuation of the biological activity of this therapeutic agent warrants further consideration.

MOFs are porous crystalline materials composed of organic ligands and metal ions/clusters linked by coordination bonds [117]. Due to their exceptional structural characteristics, such as high porosity, tunable size, chemical versatility, and flexible network topology, MOF-based drug delivery systems have gained popularity [118]. MOFs offer a rich elemental composition and adjustable porosity compared with traditional nanomaterials. Different combinations of metal clusters and organic linkers enable the MOF’s diverse properties [119]. MOFs’ structure and composition are controllable, allowing for metal nodes and various organic ligands to be adjusted. These distinctive properties make MOFs suitable candidates for various drug-delivery applications. Wang et al. designed a Cu/Zn bimetallic MOF nanoplatform (DNAzyme@Cu/zeolitic imidazolate framework [ZIF]-8) that can encapsulate therapeutic DNAzyme for gene therapy [116]. DNAzyme@Cu/ZIF-8 can release Zn^2+^, Cu^2+^, and DNAzyme in an acidic microenvironment after uptake by tumor cells, thus exerting chemotherapy and inhibiting cancer cells proliferation and metastasis (Fig. 3B). Notably, a multitude of MOF-based nanomaterials have been synthesized and applied in combating pulmonary diseases [110].

#### Other NPs

In addition to nanomaterials, extracellular vesicles are frequently used to encapsulate and deliver diverse biological substances, including microRNAs (miRNAs), mRNAs, and proteins [120, 121]. These vesicles exhibit a double-membrane structure with diameters ranging from 40 nm to 1,000 nm [122]. They can be categorized into microvesicles, exosomes, and apoptotic vesicles according to generation process, release pathway, size, content, and function [123]. Among these, exosomes with 30 to 150 nm stand out as nano-sized vesicles featuring a complete membrane structure [124]. Exosomes play a key role in intercellular signal transduction via their cargo, which predominantly comprises deliverable nucleic acids, proteins, lipids, and metabolites [125, 126]. Upon uptake by a recipient cell, diverse biomolecules contained within the exosomes are transferred, potentially triggering reactions and altering the function of the recipient cell [127]. This property has attracted considerable attention for its therapeutic applications, particularly in RNA delivery [128]. Exosome-associated gene delivery has demonstrated therapeutic effects against lung diseases [129, 130].

Moreover, peptides can be integrated into gene delivery systems as functional components to overcome biological barriers [131]. Cell-penetrating peptides (CPPs), characterized by their concise nature and containing no more than 30 amino acids, exhibit the potential to traverse biological membranes and facilitate the delivery of an assortment of biologically active compounds into cells [132]. CPPs are versatile carriers suitable for transporting siRNA, small molecules, proteins, and other in vitro and in vivo peptides that have been extensively researched [133].

## Nanomaterial-assisted gene editing in the treatment of pulmonary diseases

Nano-delivery systems have unequivocally demonstrated efficacy in targeted drug delivery for lung disease treatment that optimized the therapeutic agent administration through regulated release mechanisms, thereby increasing bioavailability [134]. To further actualize comprehensive and nuanced lung-targeted therapeutic effects, researchers are actively investigating the amalgamation of the unique attributes of nanomaterials with gene editing technologies to achieve genome modifications that are both more potent and accurate [135–137]. This synergy amplifies the precision of gene editing, mitigates unintended genetic alterations, and curtails potential adverse effects. The controlled and precise drug delivery properties based on nanomaterials have the potential to transform the landscape of gene therapy, thereby ushering in novel approaches for genetic and multifaceted diseases treatment.

### Gene editing tools

Gene editing, a transformative technology capable of precisely modifying an organism’s genetic sequence [138], achieves the precise and efficient trimming, cutting, replacement, and insertion of DNA or RNA sequences to manipulate gene expression, thereby durably affecting protein sequences or structures. With the advancement of science and technology, gene editing technology has evolved from the first generation of zinc finger nucleases (ZFN), the second generation of transcription activator-like effector (TALE) to the current third generation of CRISPR-Cas [15, 139]. CRISPR-Cas technology is the mainstream technology for gene editing due to its high efficiency, fast operation and accurate results. The traditional CRISPR/Cas system can use homology-directed recombination to perform precise editing, but the shortcomings are extremely low efficiency and error-prone, which limits its popularization and application. Currently, much research is devoted to modification and optimization to reduce the occurrence of mutations. Therefore, new editing tools based on CRISPR/Cas9, Base Editing (BE) and Prime Editing (PE) technologies have improved gene editing [140, 141]. These gene editing tools have accelerated progress in scientific discovery and opened the possibility of addressing numerous genetic disorders.

#### CRISPR/Cas system

The CRISPR/Cas system is a bacteria-acquired immune system, through which bacteria defend themselves by selectively identifying invading exogenous DNA and utilizing Cas proteins for cleavage [142]. It brings an adaptable and easy method for gene editing. It can substantially reduce the expenditure and time involved in constructing mammalian gene-editing tools [143]. This is achieved by targeting a sgRNA, which requires adjustments within the initial 20 bases to recognize diverse locations. The CRISPR motif predominantly comprises a collection of CRISPR/Cas genes and a distinctive CRISPR array. CRISPR arrays were first identified in 1987, when they were found to flank the *Escherichia coli* isozyme conversion of alkaline phosphatase (*iap*) gene sequence [144]. These arrays consist of repetitive sequences interspersed with variable sequences (spacers) targeting foreign genes. CRISPR/Cas genes typically encode Cas proteases, notably the CRISPR-related endonuclease Cas9 [145].

The Cas9 protein comprises two distinct nuclease domains, namely histidine and asparagine HNH domain and RuvC-like domain [146]. The HNH domain cleaves the non-targeted strand that complements the sgRNA, whereas the RuvC domain cleaves the intended strand. sgRNAs originate from transactivating CRISPR RNAs (tracrRNAs) and CRISPR RNAs (crRNAs). Each crRNA encompasses a conserved repeat sequence complementary to tracrRNA and a 20 nt transcribed spacer region that matches the exogenous DNA sequence. In conjunction with tracrRNAs, these crRNAs assemble with Cas9 proteins forming a CRISPR/Cas9-sgRNA complex that creates double-strand breaks (DSBs) at target sites in the genome [147]. Considering the CRISPR/Cas9 system attributes and functionalities, crRNA and tracrRNA were amalgamated into a single sgRNA, facilitating efficient DNA cleavage, and rendering the CRISPR/Cas9 system amenable for cellular and animal applications.

The CRISPR/Cas9 system effectively identifies and cleaves exogenous DNA or RNA with the appropriate specific sequence [148]. Upon the introduction of exogenous DNA into bacterial cells, the Cas1-Cas2 complex discerns protospacer adjacent motifs (PAMs) within its sequence and selectively cleaves genes proximal to the target site [149]. This initial cleavage by the Cas1-Cas2 complex triggers integration of the novel protospacer sequence into the CRISPR motif, marking the adaptation phase. Subsequently, the expression phase transpires under the influence of the oxygen ribonuclease (RNase) III and the Cas9 protein. Following the import of the prototype spacer sequence into the CRISPR sequence, the bacterium transcribes it into a precursor transcript RNA. It is then processed into mature crRNA (sgRNA) through the action of RNase III. Cas9 protein and sgRNA coalesce to form Cas9 RNP. The final crRNA synthesis yields a single spacer sequence that corresponds to the original spacer sequence of a particular exogenous DNA, signifying the onset of the interference phase [150].

During subsequent encounters with exogenous DNA, recognition of the prototype spacer sequence can occur via two distinct mechanisms: first, the sgRNA within the RNP dictates the specificity of cleavage through its alignment with the prototype spacer sequence of the target DNA; second, the Cas9 protein identifies the PAM sequence on the exogenous DNA. Eventually, the RNP, featuring the Cas9 HNH and RuvC endonuclease domains, executes DNA cleavage. This cleavage induces DSBs in the target DNA, thereby disrupting gene expression. DSBs are repaired through non-homologous end-joining (NHEG) or homologous recombination repair (HDR) [151], which is predominantly employed by cells for the swift rejoining of broken DNA strands; however, this repair process is stochastic, often leading to base insertions or deletions and consequent mutations that alter the function of the gene [152].

Furthermore, in cases where a small fragment of donor DNA with ends identical to the damaged sequence is introduced, HDR leverages donor DNA as a template, facilitating accurate repair. This repair process enables deliberate gene insertion or deletion [153]. CRISPR-Cas9 gene editing relies on custom-designed sgRNAs to guide the Cas9 protease to efficiently cleave the double-strand DNA in the target sequence. Subsequent repair mechanisms result in gene knockouts or knock-ins, thereby facilitating genome modification [154]. Currently, the CRISPR/Cas9 system is rapidly emerging as a preeminent gene editing technology that is generally employed in biomedical research to address human genetic diseases.

Ensuring secure and efficient conveyance of the gene-editing tool to the intended cell is imperative in all gene-editing methodologies. CRISPR/Cas9 systems are commonly used to deliver recombinant viral vectors or plasmids that encode DNA sequences [155]. However, the introduction of exogenous DNA carries the risk of permanent genome recombination, thereby potentially jeopardizing endogenous gene integrity and amplifying safety concerns associated with clinical applications. Furthermore, the prolonged activity of the CRISPR/Cas9 system in the cell after gene editing results in unintended off-target effects and mutations. To optimize CRISPR/Cas9 delivery, direct administration of Cas9 protein and sgRNA has become a promising strategy [156]. This approach has significant clinical potential. However, maintaining the intracellular stability of Cas9 proteins and sgRNAs to facilitate their translocation to the nucleus is challenging.

#### Base editing (BE)

Owing that the CRISPR/Cas9 system often requires the initiation of intracellular self-repair mechanisms by introducing DNA DSBs, deletions, substitutions, insertions, and other modifications are introduced during the repair process; thus, the editing results are highly randomized. To achieve the edition of a specific base more precisely, in 2016, David Liu’s team further developed a base editing technology using CRISPR [157]. This technology modifies a single base in the genome of living cells through a reliable method. Since single-base mutations cause many hereditary diseases, BE has become a powerful tool for treating many single-base genetic diseases.

BE is the fusion of Cas proteins that have lost their catalytic activity (deactivated Cas, dCas) or that cut only one strand (such as the nickase Cas, nCas) with deaminase enzymes that act on single-stranded DNA (ssDNA) to achieve base substitution at the target site [158]. Currently, base modification enzymes can be divided into cytosine base editor (CBE) and adenine base editor (ABE). The key components of CBE are nCas9 or dCas9 and cytosine deaminase. The Cas9 protein, cytosine deaminase, and uracil DNA glycosylase inhibitor (UGI) form the CBE fusion protein. The working principle is that when the fusion protein is targeted to genomic DNA by the sgRNA, the cytosine deaminase binds to the ssDNA at the R-loop region formed by the Cas9 protein, sgRNA, and genomic DNA, deaminating the cytosine (C) to uracil (U) within a specific range on this ssDNA. This converts U to thymine (T) through DNA replication or repair, directly substituting C-G to T-A base pairs. UGI binds to U and inhibits uracil N-glycosylation (UNG) to remove U, increasing the efficiency of C to T conversion on the DNA strand. The principle of ABE is similar to that of the CBE system: an adenine deaminase deaminates a certain amount of adenine (A) at the target site to inosine (I), which is read and replicated as guanine (G) at the DNA level, performing the A-G change [159]. The development of ABE surpasses the limitations of previous base editing systems that only edit C or G, providing more options for single-base mutations.

The development of the BE technology is crucial for the targeted correction of base mutations and the generation of key nucleotide variants in the genome. BE therapies have been shown to extend the lifespan of mice with progeria. Thus, a base-editing approach is a promising genetic diseases therapeutic strategy. However, BE systems still have drawbacks, such as the inability to edit all bases arbitrarily, off-target effects, insertional deletions, and bystander editing effects.

#### Prime editing (PE)

PE is a novel high-precision gene-editing technology based on the CRISPR/Cas9 system, also developed by David R. Liu’s team [160]. PE’s versatility enables the substitution, deletion, and insertion of target gene sequences, making it a promising treatment strategy for a broad range of human genetic diseases.

The components of PE consist of a nCas9 fused with a reverse transcriptase (RT) and a pilot editing guide RNA (pegRNA) that directs nCas9 to the target site and serves as a template to guide the RT for editing [161]. First, guided by the pegRNA, nCas9 cleaves the PAM-containing target DNA strand. The cleaved target DNA strand is complementary to and binds to the sequence of the 3’-end primer binding site (PBS) of the pegRNA, after which the reverse transcriptase acts on the reverse transcription template sequence. At the end of the reaction, 5’- and 3’-flaps structures are formed in dynamic equilibrium at the target site of the DNA strand, where the DNA strand of the 3’-flap structure carries the target mutation while that of the 5’-flap structure is free of any mutation. The 5’ flap structure is easily recognized and excised by structure-specific endonucleases, and then the DNA ligation and repair perform precise gene editing. Pilot editing has been applied to various cell types, organoids, mouse embryos, and plants with high accuracy and precision. However, compared to previous generations of gene editing methods (e.g., Cas9 and base editing), the efficiency of PE is still very low and varies widely from one editing site to another.

In summary, the advances in gene editing enabled by the CRISPR-Cas system have paved the way for the development of basic research and therapeutic applications. However, to fully realize the potential of gene editing, the systems must be delivered safely and efficiently to specific tissues in vivo.

### CRISPR/Cas9 system delivery

Regardless of whether it is applied to in vitro or in vivo gene editing scenarios, secure and efficient conveyance of the CRISPR/Cas9 system to the designated site is a fundamental requirement for its operational success. Presently, three primary delivery methodologies for CRISPR/Cas9 have been recognized, namely the introduction of plasmids encompassing the Cas9 protein and sgRNA-encoding sequences, administration of Cas9 protein mRNA and sgRNA, and direct administration of Cas9 protein and sgRNA [147].

#### Delivery of plasmids encoding Cas9 proteins and sgRNAs

The delivery of plasmid DNA (pDNA) containing Cas9 protein and sgRNA sequences facilitates sustained and stable intracellular expression of these elements, including HDR repair templates [155]. Notably, double-stranded DNA is a commonly employed delivery format because of its stability and ease of manipulation compared with Cas9 protein and mRNA. Various physical methods, including electroshock and injection, are well-suited for this approach. However, pDNA’s substantial size and pronounced negative charge present challenges to loading and encapsulation, considerably increasing the complexity of delivery and expression with the CRISPR/Cas9 system [162]. In prokaryotes, the endogenous CRISPR/Cas system is a coping strategy. Some bacteria and archaea have their own CRISPR/Cas systems that can be used for gene editing without exogenous nucleases. For example, the recent development of an editable protein delivery system by Feng Zhang’s team promises to deliver any protein precisely to the target cell [163]. In addition, the stable presence of nanomaterials in circulation and their ability to accumulate at specific sites in vivo could enhance the therapeutic effects of CRISPR gene-edited drugs. Yan et al. used a complex of the cationic polymer poly(β-amino ester) (PBAE) with a plasmid encoding the CRISPR system at the core, combining targeted delivery and conditional activation of CRISPR-Cas9 for precision therapy of inflammatory bowel disease [164]. Furthermore, hybrid exosomes generated by liposome incubation are also new and effective strategies for drug encapsulation and in vivo delivery of the CRISPR-Cas9 system [165].

#### Delivery of Cas9 protein mRNA and sgRNA

The delivery of Cas9 protein mRNA and sgRNA involves the initial transcription of Cas9 protein mRNA in vitro, followed by its co-transfer into target cells along with sgRNA. Upon introduction into target cells, Cas9 mRNA and sgRNA are translated, resulting in the production of Cas9 protein. Subsequently, Cas9 protein and sgRNA combine within the cell to form RNP complexes, facilitating their role in gene editing [155]. Notably, during gene editing, Cas9 mRNA’s nuclear entry is unnecessary, and its translation and processing into Cas9 protein occur within the cytoplasm. The transient nature of Cas9 protein expression decreases off-target effects compared to CRISPR/Cas9 plasmids, thereby enhancing efficacy. Cationic liposomes serve as the primary vehicles for mRNA delivery [166]. However, this delivery approach is constrained by Cas9 mRNA’s short cytoplasmic half-life, necessitating more robust delivery systems. Furthermore, in vivo-synthesized RNA triggers an immune response via pattern recognition receptor activation. Recent research indicates that judicious chemical modification of mRNAs not only confers stability and prevents degradation but also mitigates immune responses [167].

#### Delivery of Cas9 protein and sgRNA

The most effective strategy for utilizing CRISPR/Cas9 system is the direct delivery of Cas9 protein and sgRNA. This direct approach bypasses transcription and translation process, resulting in rapid gene editing and enhanced application potential [168]. Incubation of Cas9 protein and sgRNA in vitro produces an RNP complex, which is rapidly degraded upon entry into the cell and acts quickly. This transient functionality diminishes off-target effects and toxicity. Notably, the Cas9 protein (~ 160 kDa) has a large molecular mass and a positive charge, whereas the sgRNA has a pronounced negative charge [169]. This charge disparity complicates the efficient delivery of Cas9/sgRNA RNPs. When Cas9 RNPs are displayed on target cell surface, anti-Cas9 T cells may initiate an attack against these cells. Consequently, the focus of designing and crafting delivery systems must be on preserving Cas9 nuclease activity while shielding RNPs from recognition by proteases, antibodies, and T cells in bodily fluids [170]. Augmentation of the delivery system with CPPs or NLS further facilitates RNP translocation into the nucleus for functional execution.

### CRISPR/Cas9 system delivery method

The CRISPR/Cas9 system requires a specific delivery mechanism to penetrate an organism and execute its editing function. The efficiency of delivering the editing system to the organism directly affects the efficiency of gene editing. Delivery vectors can be categorized into physical delivery, viral, and non-viral vectors [171].

#### Physical methods

Physical delivery entails the use of devices to directly transport the CRISPR/Cas9 system into the cytoplasm or nucleus, facilitating rapid cleavage of the targeted DNA. Delivery techniques include electroporation and microinjection. The Cas9-sgRNA complex encoded within the plasmid traverses the cell membrane through microinjection. Schumann et al. employed an electroporation technique to transport Cas9/sgRNA RNP into CD4^+^ T cells, achieving a remarkable 40% gene editing efficiency [172]. This transient Cas9/sgRNA RNP delivery has significant advantages compared with the ablation of cell surface markers in human CD4 T cells by transfection of Cas9/sgRNA plasmids as reported by Mandal et al. [173]. This is because the Cas9/sgRNA plasmid ablated cell surface markers in human CD4 T cells at a significantly lower efficiency compared with other cell types. Possible reasons for this are the suboptimal Cas9 or sgRNA levels or Cas9-RNP complex formation. Cas9/sgRNA SNP-based delivery bypasses these drawbacks. This work established a broadly applicable approach for genetic manipulation of human primary T cells. However, physical delivery methods that rely on mechanical action exhibit heightened cytotoxicity and require specialized equipment, rendering them impractical for in vivo therapeutic applications.

#### Viral vectors

Viral vectors are the current preferred mode of delivery for the CRISPR/Cas9 system. This approach involves the encapsulation of DNA containing nucleic acid-coding sequences within a viral structure, followed by its introduction into the target cell. Prominent viral vectors encompass lentiviral vectors, adenoviruses, adeno-associated viruses, and bacteriophages [174]. Lentiviral vectors derived from human immunodeficiency virus 1 have a spherical structure containing single-stranded RNA and are extensively utilized for CRISPR/Cas9 delivery due to their remarkable ability to accommodate up to 7 kb of genetic material, including the *S. pyogenes* Cas9 (SpCas9) gene and one or more sgRNAs [175]. Adenoviruses, which are non-enveloped linear double-stranded DNA viruses, exhibit broad host compatibility, genetic stability, superior transduction efficiency, and substantial loading capability compared with alternative viral vectors [176]. In contrast, adeno-associated viruses demonstrate diminished immunogenicity, prolonged sequence persistence in nondividing cells, stable transgene expression, favorable safety profiles, and therapeutic potential. Consequently, adeno-associated viruses are the foremost choice for CRISPR/Cas9 system delivery and have been approved as gene-enhanced therapies in numerous human clinical trials [177]. Bacteriophage vectors, primarily used against multidrug-resistant bacteria, have limited research applications because of their narrow host specificity [178]. Although viral vectors offer high transfection efficiency, their proclivity for mutations and carcinogenic risks presents substantial challenges for clinical applications. Additionally, limited loading capacity makes efficient CRISPR/Cas9 delivery with adeno-associated viruses difficult.

#### Nonviral vector

Non-viral vectors include synthetic materials that facilitate gene transfer [179]. In contrast to physical delivery methods and viral vectors, non-viral vectors have low immunogenicity, which is crucial for preserving the physicochemical stability of cargos and preventing enzymatic degradation both extracellularly and intracellularly [180]. These properties can be obtained by integrating chemical modifications in various CRISPR/Cas9 vectors and by utilizing various biomaterials to formulate effective and secure delivery systems [181].

Among non-viral vectors, lipids, polymers, organic or hybrid NPs, and CPPs are the most frequently used [182]. These vectors have demonstrated the capacity to deliver CRISPR/Cas components proficiently and safely in vivo to a wide range of target genes across various tissues [183, 184]. As Cas9 proteins carry positive charges, LNPs need to be modified to deliver Cas9-gRNA RNPs, whereas delivery based on CRISPR/Cas9 plasmids and mixtures of gRNAs and Cas9 mRNAs do not require significant modification. LNPs present several advantages, including simple preparation, safety in target cells, and small off-target effects in CRISPR/Cas9 delivery, however, their transduction efficiency is low [185, 186]. To improve the delivery efficiency, Kang et al. utilized cationic polymers to covalently modify polymer-derived Cas9 proteins directly for subsequent complexation with single-conductor RNA targeting antibiotic resistance [187]. This nanoscale CRISPR complex maintained Cas9 endonuclease functional activity to induce double-stranded DNA cutting. This approach has great potential for improving delivery efficiency compared with liposomes. In addition, Cas9 proteins interact with non-arginine-based CPPs, and gRNA complexes electronically couple to CPPs via covalent coupling to form NPs with a positive charge. These NPs can readily pass through cell membranes, resulting in CRISPR/Cas9 efficient delivery.

Although nanomaterial-based delivery systems are increasingly gaining attention for gene therapy, the safety and efficiency of CRISPR/Cas9 nanocarriers pose significant challenges due to various in vivo barriers. The main challenge is to efficiently encapsulate the CRISPR/Cas9 components. For example, the CRISPR/Cas9 plasmid (pX330) is a single plasmid encoding the Cas9 nuclease and sgRNA, with a molecular weight of 5.55 × 10^3^ kDa and a negative charge of 1.74 × 10^4^ C [188]. SpCas9 mRNA is approximately 4,300 nt long and contains 4,300 negative charges [189]. For a large Cas9 protein (160 kDa), the RNP has a size of approximately 10 nm and contains a negatively charged sgRNA [190]. It is difficult to concentrate the CRISPR/Cas9 system in a single delivery vehicle, owing to its large volume and electrically charged surface. Zou et al. developed a CRISPR-Cas9 nanocapsule for targeting brain tumor cells, enabling a gene therapy strategy for malignant glioma [191].

In conclusion, an effective delivery is crucial for the CRISPR/Cas9 system to achieve the desired editing efficiency. However, various nanocarriers achieve the effective protection and targeted delivery of the CRISPR/Cas9 system. The synergistic application of different nanocarriers greatly enhances the editing efficiency, blood circulation, intracellular uptake, and intracellular transport and localization of Cas9 and sgRNA. In addition to the efficacy, the toxicity of the delivery systems should also be considered. When designing nanomaterials, attention must be paid to their degradability, overall charge density, topology, and size. Although non-viral gene vectors have less immunotoxicity than viral vectors, it should not be overlooked that nanoparticles can induce host immune responses. Therefore, the delivery method of mRNA and Cas9 RNP allows for a short retention in the host cell. In summary, the ideal vector must meet the requirements of efficacy, safety, and controllability. Although most CRISPR/Cas9 nanocarriers do not meet all the requirements for clinical trials, the prospect is certainly positive. The continued research efforts in various fields will eventually surpass any limitations.

## Advances in nanomaterial-assisted gene editing for optimizing the treatment of pulmonary diseases

Nanomaterial-based carrier systems exhibit controllable structure, efficacious functionality, low immunogenicity, and potential for mass production [192]. Compared to conventional gene delivery methods, functionalized nanomaterial delivery vectors can reduce the cytotoxicity of freely diffusing gene vectors, limit ectopic expression of transgenes in neighboring tissues, improve gene vector stability, and control gene transfer and expression level [193]. Consequently, these nanomaterial-mediated genome editing techniques have considerable potential for treating congenital or immunological pulmonary disorders.

### Lung cancer

Lung cancer can be roughly categorized into small cell lung cancer (SCLC) and non-small cell lung cancer (NSCLC). SCLC is an aggressive neuroendocrine tumor characterized by its propensity for recurrence, limited therapeutic options, and poor prognosis. NSCLC accounts for about 80% of lung cancers, grows and divides slowly, and spreads relatively late compared with SCLC [194]. Despite sustained efforts and advancements in lung cancer treatment and diagnosis, the overall survival rate for lung cancer remains low.

The primary goal of cancer therapy is to impede tumor growth and progression through the targeted correction of mutations and restoration of dysregulated gene expression, including that of oncogenes, chemotherapy-resistant genes, metabolism-associated genes, and genes associated with tumor stem cells. Lung cancer, a type of cancer with a multitude of causative oncogenic mutations, is a salient example. Notably, enhanced expression of the epidermal growth factor receptor (*EGFR*) gene is closely associated with NSCLC. Overexpression of *EGFR* on the surface of NSCLC cells promotes tumor advancement and decreases the survival prospects of individuals afflicted with lung cancer, and chemical inhibitors of EGFR have been used as first-line treatments for *EGFR*-mutant NSCLC. Individuals’ genomes have been modified by CRISPR/Cas9-mediated genome editing to treat lung cancer patients with *EGFR* mutations. For example, Han et al. constructed a multifunctional delivery vector modified with AS1411-coupled hyaluronic acid and NLS-GE11 peptide-coupled hyaluronic acid to deliver an *EGFR*-knockout CRISPR-Cas9 plasmid [195], which effectively blocked the fusion of A549 lung cancer cells. In addition, the authors demonstrated specific delivery of the genome editing plasmid to circulating malignant cells (CMCs) in blood samples from cancer patients to knock down *EGFF*, highlighting the inhibitory effect of *EGFR* knockdown on cell fusion. Although conducted in vitro, this study provides valuable insights for dynamically adjusting and optimizing cancer treatment through timely and accurate assessment of treatment efficacy in individual tumor patients, thus providing a novel and effective platform for personalized precision therapy.

**Fig. 4:**
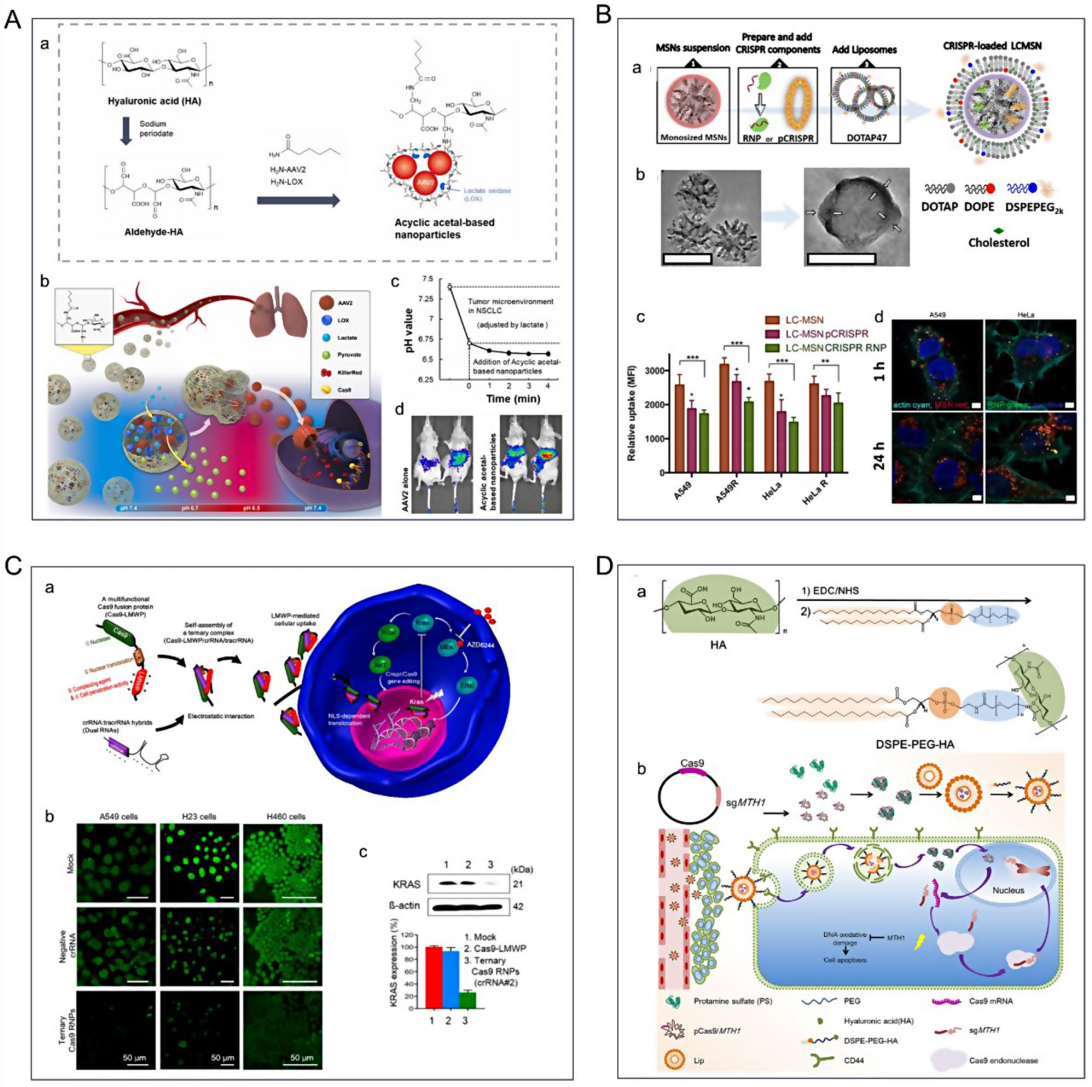
(**A**) Acyclic acetal-based nanoparticles for viral therapy of NSCLC [198]. (a) Synthetic route of acyclic acetal-based nanoparticles. (b) Targeted delivery of acyclic acetal-based nanoparticles. (c) Effect of pH conditions of tumor microenvironment on acyclic acetal-based nanoparticles. (d) In vivo luminescence images of luciferase after injection of acyclic acetal-based nanoparticles or adeno-associated virus serotype 2. (**B**) Lipid-coated MSNs for CRISPR delivery [199]. (a) Synthetic route of CRISPR@LC-MSN. (b) Transmission electron microscopy characterization of MSNs and RNP@LC-MSNs. (c-d) Cellular uptake of LC-MSN. (**C**) A carrier-free ternary Cas9 RNP delivery system for in vitro and in vivo gene editing [200]. (a) Ternary Cas9 RNP delivery system for *KRAS* treatment in NSCLC. (b) *KRAS* expression in different cells after delivery of three Cas9 RNPs. (c) Ternary Cas9 RNPs inhibited KRAS expression. (**D**) PS@HA-Lip for targeted delivery of mutT homolog1 plasmid (pMTH1) for NSCLC therapy [201]. (a) Synthetic route of DSPE-PEG-HA. (b) Mechanism of PS@HA-Lip/pMTH1 for NSCLC therapy. Reprinted with permission from Ref [198–201]

In addition, a significant factor affecting lung cancer patient survival is resistance to targeted molecular drugs. Research indicates that this resistance is closely associated with the constant mutation of oncogenic genes, which are potentially linked to the production of lactic acid during tumor glucose metabolism. In particular, mutations in the *EGFR*, Kirsten rat sarcoma virus oncogene homologue (*KRAS*), and anaplastic lymphoma kinase (*ALK*) have been identified as causing the upregulation of lactic acid secretion, consequently lowering the pH of the tumor microenvironment [196]. Various therapeutic approaches have emerged for lung cancer treatment [197], including the utilization of acid-degrading NPs along with existing lysosomal virus therapies. Tseng et al. used lactic acid secreted by NSCLC tumors to create acid-degrading nanoparticles, incorporating an acyclic acetal component of oxidized hyaluronic acid (HA) for the release of recombinant adeno-associated virus serotype 2 [198]. This involves the binding of adeno-associated virus serotype 2, lactate oxidase, and hexanoamide to the HA aldehyde through reductive amination (Fig. 4A). Acyclic acetal-based NPs can effectively reduce lactate levels within the extracellular microenvironment of NSCLC, leading to a localized pH reduction. Since the proteases of the viral capsid are pH-sensitive, a decrease in pH facilitates the internalization of the virus within the cell. The experimental findings suggest that employing acyclic acetal-based NPs in an NSCLC model can target and control carrier delivery and enhance Cas9 protein expression. The confirmation of site-specific viral transduction within the LA microenvironment of NSCLC tumors underscores the potential of this approach to enhance outcomes in general NSCLC therapy or cases of drug-resistant NSCLC. Noureddine et al. prepared a mono-sized lipid-coated MSN carrier loaded with Cas9 RNP as shown in Fig. 4B, which was efficiently released within tumor cells (70%) [199]. The editing efficiencies of RNP@LC-MSN ranged from 46 to 60% in A549 lung cancer cells, and 10 ± 2% in localized tissue regions *in mice*.

Kim et al. designed a carrier-free ternary Cas9 RNP delivery system for robust gene editing in vitro as well as in vivo [200]. The Cas9 fusion protein was constructed using a low-molecular weight protamine (LMWP) peptide from a natural source of CPP coupled to Cas9, carrying both NLS and LMWP. NLS mediates the localization of its nucleus for functional editing, and LMWP enables self-assembly of the ternary complex (Cas9-LMWP/crRNA/tracrRNA) through electrostatic-driven interactions and cellular internalization (Fig. 4C). The ternary complex-induced KRAS disruption effectively inhibited the growth of human NSCLC cells. Wang et al. designed a multifunctional non-viral carrier capable of targeted delivery of Cas9/sgMTH1 plasmids (pMTH1) to tumor cells **(**Fig. 4D**)** [201]. The concentration of pMTH1 with protamine through electrostatic interactions and control of the ratio of protamine sulfate to the plasmid yielded negatively charged complexes. To prevent nuclease degradation in the bloodstream, protein/DNA complexes were encapsulated within liposomes. Further modification by doping DSPE-PEG-HA into PS@Lip/pMTH1 resulted in active targeting of tumor cells. These findings indicate that PS@HA-Lip facilitated CRISPR-Cas9 plasmids delivery into tumor cell nuclei, resulting in genome editing effects. Knockdown of MTH1 using PS@HA-Lip led to growth inhibition in NSCLC. Moreover, it promotes tumor cell apoptosis and reduces liver metastasis in NSCLC.

### Pneumonia

Pneumonia is a pulmonary disease primarily triggered by pathogens including bacteria, viruses, fungi, and other microorganisms. Although antibiotics and antiviral agents play pivotal roles in its treatment, the generation of drug-resistant strains and intricacies of the disease underscore the need for novel therapeutic modalities [202, 203]. In recent years, the amalgamation of nanomaterials using gene editing technologies has ushered in novel prospects for pneumonia treatment.

The outbreak of SARS-CoV-2 has expedited mRNA vaccine development by researchers worldwide, marking an important milestone in nanotechnology-based gene delivery from fundamental research to clinical applications. The FDA approved two mRNA vaccines from Moderna and BioNTech/Pfizer Pharmaceuticals for the prevention of coronavirus disease 2019 (COVID-19) [204]. Notably, both mRNA vaccines use LNPs as carriers for therapeutic genes [205]. However, although LNPs have achieved remarkable success as gene carriers [206], storing them for extended periods presents a challenging problem for clinical applications. Various long-term storage conditions for encapsulated mRNA LNPs have been investigated, and the stability of LNPs has been assessed by different cryoprotectant concentrations, such as mannitol, alginate, or sucrose. It was shown that adding alginate or sucrose (5%, w/v) to LNPs improves mRNA delivery efficiency for approximately three months [207].

**Fig. 5:**
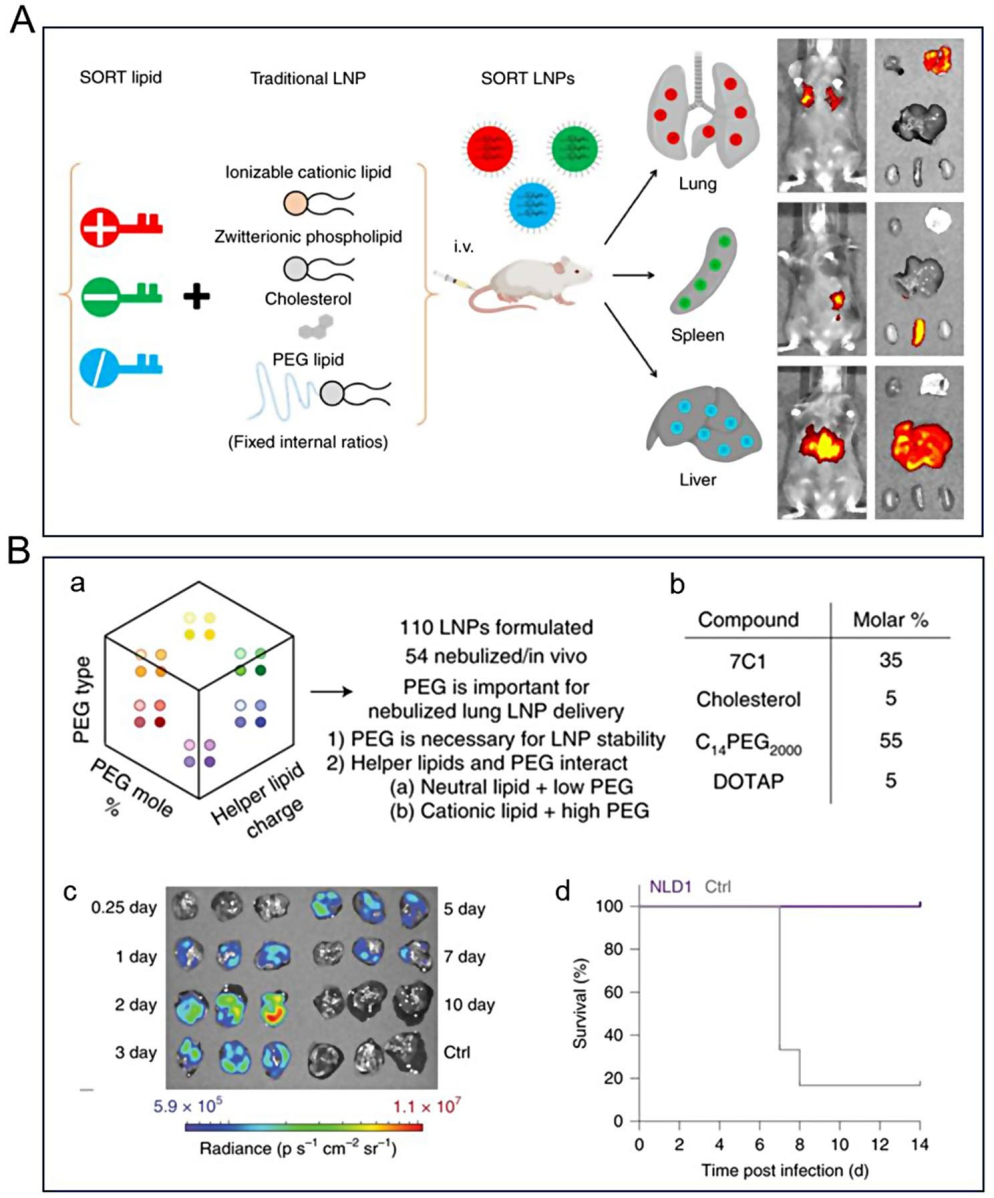
(**A**) SORT-LNP prepared by adding different SORT molecules to traditional LNP for targeting different organs [208]. (**B**) LNPs optimization for delivery of nebulized therapeutic mRNA to the lungs [209]. (a) Optimizing LNP-targeted lung delivery. (b) Mole ratio of NLD1 components. (c) Expression of NLD1 carrying AncNanoLuc mRNA in different tissues of mice. (d) Survival of H1V1-injected mice treated with NLD1 was 100%. Reprinted with permission from Ref [208, 209]

Targeting strategies are extensively employed in the design and formulation of lipid nanoparticles to enhance drug therapeutic efficacy while minimizing adverse effects. Cheng et al. devised a Selective Organ Targeting (SORT) method to modify LNPs using varying proportions of SORT molecules [208]. The authors modulated the internal charge of LNPs by adding a fifth charged liposome on top of keeping the four components (ionizable lipids, phospholipids, cholesterol, and PEG-lipids) and the corresponding ratios of conventional LNP unchanged, thereby modulating the organ targeting of LNPs (Fig. 5A). The ability of SORT LNPs to co-deliver Cas9 mRNA and sgRNA as well as deliver Cas9 RNPs was experimentally verified, successfully realizing organ-selective gene editing. ReCode Therapeutics, a clinical-stage gene therapy company, announced that its inhaled mRNA therapeutic programs based on SORT LNPs technology, RCT1100 and RCT2100, had entered clinical trials for the treatment of primary ciliary dyskinesia (PCD) and cystic fibrosis (CF). In an efficacy validation study using a PCD model based on human bronchial epithelial (hBE) cells, SORT LNP-formulated DNAI1 mRNA delivered as an aerosol successfully rescued ciliary function for weeks after the last treatment. These data suggest that the SORT LNP delivery platform offers new approaches to gene therapy for rare and common genetic diseases. In addition, Dahlman et al. designed LNPs for effective drug delivery and effectively targeted delivery of therapeutic mRNAs to the lungs by nebulizing the LNPs [209]. The authors optimized the composition, molar ratio, and structure of LNPs made from lipids, helper lipids, and PEG, and investigated the in vivo workflow of LNPs for mRNA delivery to the lungs after nebulization (Fig. 5B). The optimal ratio of nebulized lung delivery 1 (NLD1) vectors was screened; they can be used to target mRNAs delivering a broad range of neutralizing antibodies to protect mice from the lethal challenge of H1N1 influenza.

Polymer NPs have been employed in gene therapy for lung diseases. For example, poly (β-amino ester) was combined with PEG-lipids to obtain an mRNA carrier with stability and good efficacy. By intravenous injection, the carrier was able to successfully deliver mRNA to the lungs of mice and the efficacy of degradable lipid polymer NPs for systemic mRNA delivery was confirmed [210, 211]. In addition, macrophage-specific gene editing was achieved using CRISPR/Cas9 components delivered by PEG-b-PLGA-based cationic lipid-assisted NPs [212, 213].

In addition to acting as gene carriers and targeting agents, NPs play a pivotal role in facilitating gene editing techniques to investigate resistance against pneumonia pathogens. Precise genetic modification of these pathogens can effectively mitigate or eliminate their resistance to antibiotics. Moreover, NPs are widely recognized as valuable tools for enhancing antimicrobial agents’ delivery. Specifically, NPs leverage two primary mechanisms to combat bacteria effectively: (i) the disruption of membrane potential and integrity and (ii) the induction of oxidative stress via nanoparticle-catalyzed reactive oxygen species production. These mechanisms operate both independently and synergistically [214].

CRISPR/Cas system is an effective tool to control antibiotic-resistance gene prevalence in bacteria and to eradicate pathogens with remarkable precision. For instance, *Klebsiella pneumoniae* often develops resistance to antibiotics, such as mucin and tigecycline, owing to chromosomal gene mutations [215]. Recent advances in CRISPR/Cas9-based gene editing methods have enabled *K. pneumoniae* segmentation gene *parA* disruption and the cleavage of the prevalent carbapenemase resistance plasmid [216]. Nevertheless, despite significant strides in the NP-based CRISPR/Cas delivery, the integration of NPs and the CRISPR system remains in the early stages of development. Successful applications in treating bacterial infections and controlling antimicrobial-resistant bacteria represent a considerable research challenge [210].

### COPD

COPD is a prevailing and debilitating chronic respiratory disease featuring obstructive bronchitis, emphysema, and persistent lung inflammation, culminating in an irreversible restriction of airflow. COPD emerges due to the complex interplay between environmental exposure and genetic predisposition [217]. Effective prevention and management of chronic respiratory disorders have garnered global attention. A primary obstacle to alleviating these ailments, particularly COPD, is the lack of effective pharmaceutical intervention. Several drugs, including antibiotics, bronchodilators, and glucocorticoids, are used in the clinical treatment of COPD. However, although these agents offer therapeutic benefits, they lack specificity. Furthermore, the conventional modes of drug administration are fraught with challenges related to their efficacy and efficiency.

NPs delivery has the potential to increase drug concentrations in the lungs while decreasing systemic adverse effects. Li et al. successfully engineered controlled-release NP drug carriers incorporating black phosphorus quantum dots (BPQDs) using an ionic crosslinking method [218], which was achieved by combining chitosan with BPQDs via surface modification with PEG (PEG@CS/BPQDs-AM NPs). In this study, the hydrophilic region of PEG and the positive charge of chitosan facilitated the penetration of these nanocarriers through the mucus layer and their adhesion to epithelial cells. Furthermore, the oxidative degradation of BPQDs led to protonation of the amino groups in chitosan, enhanced antimicrobial properties of chitosan and prevented biofilm formation. Consequently, these carriers exhibited enhanced capabilities to traverse the lung mucus barrier and facilitate drug delivery, yielding a synergistic benefit in the context of COPD. This study introduced a novel therapeutic approach aimed at addressing the challenge of suboptimal drug treatment stemming from the mucus barrier in respiratory diseases.

The identification of disease-triggering pathways and gene targets associated with COPD has led to growing interest in the investigation of miRNAs and synthetic siRNAs as potential therapeutic options. Saleem et al. developed NPs incorporating the cationic lipid 1,2-dioleoyl-3-trimethylammonium propane (DOTAP) for miR-146a targeted delivery to attenuate interleukin-1 receptor-associated kinase-1 gene expression in COPD patients [219]. Furthermore, various researchers have explored selective drug and gene delivery strategies using PLGA nanosystems for COPD treatment. Lokras et al. optimized a lipopolymer formulation employing lipid-like 5 and PLGA to encapsulate siRNAs targeting tumor necrosis factor (TNF)-α [220], resulting in effective gene silencing within macrophages. Frede et al. investigated a polymeric nanocarrier with a calcium phosphate core that exhibited preferential accumulation in the lungs and bronchial lymph nodes [221].

Furthermore, CRISPR/Cas-based systems have demonstrated substantial therapeutic potential in COPD. Sterile alpha motif-point domain-containing erythroblast transformation specific (Ets)-like factor (SPDEF) has been implicated in excessive mucus secretion observed in COPD patients [222]. To target epigenetically edited proteins at the SPDEF promoter in human lung epithelial cells, dead Cas9 was employed. This system suppresses SPDEF expression through DNA methylation, histone methylation modifications to the promoter, and recruitment of a transcriptional repression complex. Notably, the effect of transcriptional silencing persisted during cell division, suggesting the possibility of enduring phenotypic changes without the continuous presence of CRISPR editing constructs. In addition, CRISPR/Cas9 vectors tailored for COPD include methylation-mediated vectors directed towards genes such as interleukin (IL)-4, IL-13, and thymic stromal lymphopoietin (TSLP), and peptide-mediated cytotoxicity vectors [223]. These sophisticated tools facilitate the gene-editing precise delivery for therapeutic purposes.

### Cystic fibrosis (CF)

CF is a monogenic autosomal recessive disorder caused by mutations in a transmembrane conductance regulator (*CFTR*) gene. This condition primarily manifests as a dysfunction of the endocrine and exocrine glands, resulting in mucosal gland hyperplasia and increased secretion with heightened viscosity [224]. Early obstruction of mucosal secretions may precipitate pulmonary atelectasis and secondary and recurring infections, culminating in progressive pulmonary fibrosis and obstructive emphysema. Ultimately, these complications cause respiratory failure and pulmonary heart disease. The current therapeutic arsenal predominantly comprises anti-infective treatments, bronchodilators, and gene therapy. Given that mutations in *CFTR* underlie CF pathology, gene therapy holds great promise. Nonviral vectors offer distinct advantages, including lower production complexity, reduced costs, prolonged shelf life, diminished immunomodulatory responses, and enhanced drug resistance. Thus, non-viral vectors have been used to target *CFTR* genes for therapeutic purposes.

Currently, GL67A cationic lipid formulation has been utilized in CF clinical trials and is the most potent non-viral vector currently available [225], which is designed to facilitate the endosomal escape of pDNA. Studies have shown that the GL67A/pDNA complexes were stable when administered via nebulization [226]. Various other cationic lipids, including DOTMA, DOPE, and DOTAP, have been investigated for their potential as non-viral vectors in CF gene therapy [227]. In addition, a novel nonviral vector based on a cell-penetrating peptide utilizing glycosaminoglycan-conjugated enhanced transduction (GET), to facilitate effective gene transfer has been reported. To enhance the in vivo delivery potential of the GET peptide, researchers added PEG modifications to stabilize particles and sustain gene transfer activity [228]. Findings obtained using multiparticle tracking techniques demonstrated that PEG-GET complex could efficiently traverse the mucus network and rapidly disperse within sputum samples obtained from individuals with CF. Moreover, when evaluated in vivo, PEG-modified vectors demonstrated better biodistribution, biosafety, and gene transfer efficiency than unmodified vectors.

Non-viral gene therapy vectors, including cationic liposome/pDNA complexes and dense DNA NPs carrying the *CFTR* gene, have shown promise in clinical trials as potential treatments for CF. Nevertheless, data on *CFTR* expression levels in the respiratory epithelium remain insufficient and limited in duration [229]. Therefore, developing efficient and durable transgene expression strategies is urgently needed. As novel gene editing techniques emerge, correction at the chromosomal native location of the defective gene has become a viable prospect. In the case of CF, these techniques offer the capability to rectify specific *CFTR* mutations, thereby reinstating their functionality and effectively addressing the fundamental issues underlying CF. This approach diverges from the current gene therapy paradigm, which involves supplementing affected cells with a functional copy of *CFTR* [230].

**Fig. 6:**
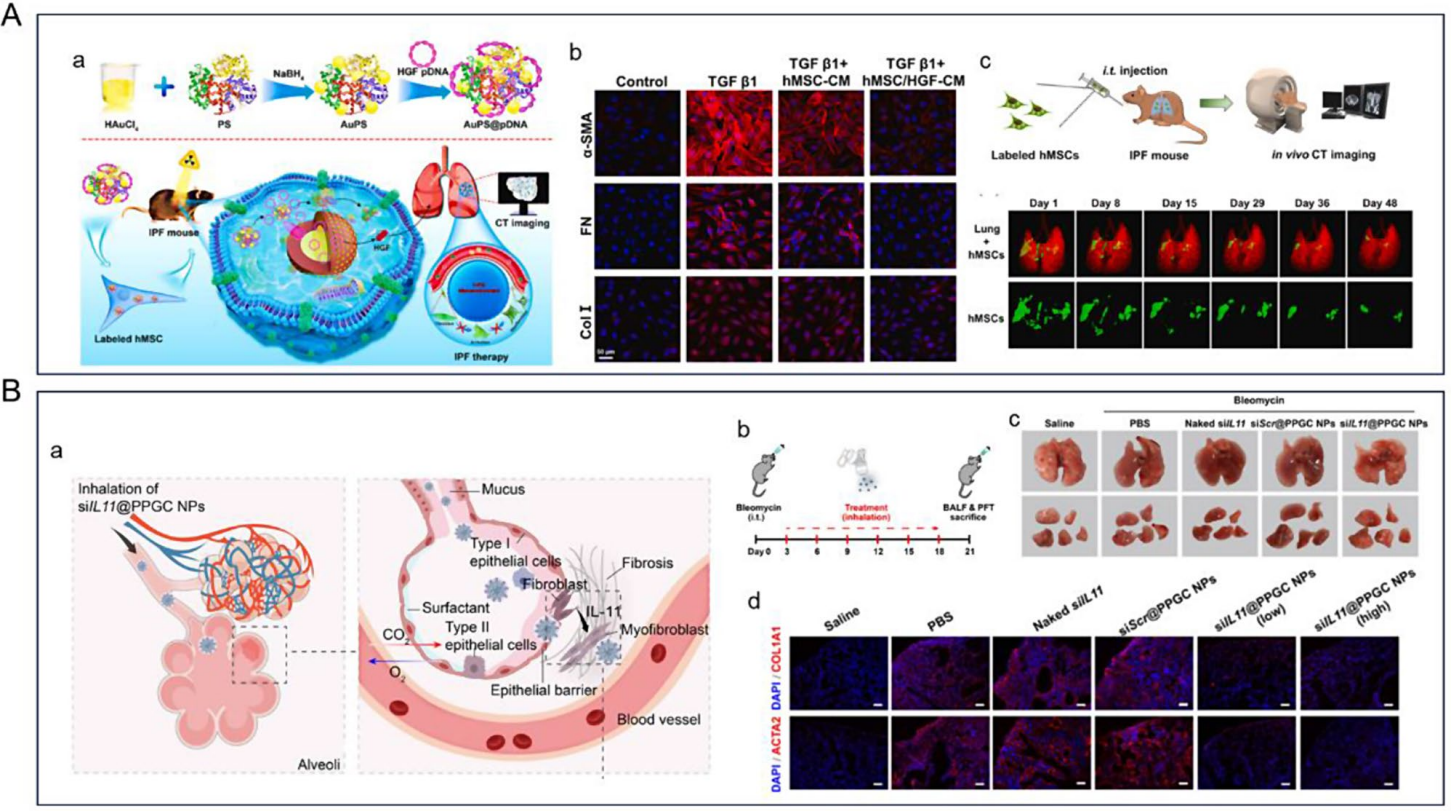
(**A**) Functionalized nano-delivery vector protamine sulfate stabilized Au NPs (AuPS)@pDNA for the treatment of IPF [231]. (a) Synthesis and therapeutic mechanism of AuPS@pDNA. (b) AuPS@pDNA-tagged hMSCs inhibit lung fibrosis in IPF cell model. (c) Three-dimensional computer tomography (CT) imaging of AuPS@pDNA-labeled hMSCs transplanted into the lungs of IPF mice. (**B**) Inhaled siIL11@PPGC NPs for the treatment of lung fibrosis [232]. (a) Inhaled siIL11@PPGC NPs into mouse lung fibroblasts for IPF treatment. (b) Inhalation therapy experimental design. (c-d) Lung tissue images (c) and immunofluorescence staining (d) of lung tissues of mice in different treatment groups. Reprinted with permission from Ref [231, 232]

Gene therapy has shown promise in clinical trials for treating CF [230]. Advances in gene therapy delivery mechanisms can provide valuable insights into the refinement of gene-editing delivery systems. For instance, Bao et al. developed an Au-based non-viral nanocarrier protamine sulfate stabilized Au NPs (AuPS)@pDNA for delivery of HGF pDNA into mesenchymal stem cells (MSCs) to improve the therapeutic efficacy of idiopathic pulmonary fibrosis (IPF). Meanwhile, as an effective CT contrast agent, it helps to elucidate the mechanism of transplanted MSCs for the treatment of IPF (Fig. 6A). This study synthesized PS-stabilized Au-based nano-delivery carriers (AuPS). HGF is a pleiotropic cytokine with promising anti-fibrotic effects and thus was delivered as a therapeutic gene into MSCs. Such engineered MSCs integrating therapy and visualization are expected to be used as novel therapeutic reagents in IPF treatment [231]. Bai et al. developed an inhalable NPs self-assembled from biodegradable PLGA-PEG copolymer and cationic lipid G0-C14, which could effectively deliver siIL11@PPGC NPs locally to the lungs of fibrotic mice, enabling the treatment of pulmonary fibrosis and significant improvement of lung function (Fig. 6B) [232]. Currently, gene manipulation techniques and molecular targets are being explored. The CRISPR/Cas9 technology for genome editing has substantial potential but remains in the nascent stages of development. In 2013, Schwank et al. achieved successful repair of the F508 mutation within the *CFTR* gene in intestinal stem cell-like organoids derived from CF patients through the utilization of the CRISPR/Cas9 system [233]. Subsequently, the viability of applying CRISPR/Cas9 technology to cystic fibrosis was firmly established.

In summary, CRISPR/Cas9 systems offer significant potential as genome-editing strategies, demonstrating their ability to achieve specific and functional correction of mutant *CFTRs in vitro* [234]. Nevertheless, the translation of these strategies into clinical practice remains a distant goal, necessitating further research and optimization efforts.

## Synthetic biology in the treatment of pulmonary diseases

Building new biological systems based on known biological systems to help humankind solve many problems in nature and social sciences has been a pursued goal. With the genomics revolution and the rise of systems biology in the 1990s, synthetic biology was developed to create, control, and program cellular behavior, and has become a major international scientific frontier [235]. As an avant-garde interdisciplinary domain, synthetic biology draws from numerous fields, including life sciences, engineering, genomics, informatics, mathematics, chemistry, and computer science [18]. Synthetic biology holds great promise for diverse applications in medicine, energy, materials, chemicals, and agriculture.

### The development of synthetic biology

The origins of synthetic biology lie in the early 1960s when Jacob and Monod made groundbreaking discoveries on the regulation of lac operons in *E. coli*, an achievement that earned them the Nobel Prize [236]. The authors found that a protein known as a transcription factor can bind to the promoter region of a gene, thereby regulating its synthesis rate by activating or repressing it. This paves the way for viewing gene expression as a dynamic system with recognizable inputs and outputs. This concept holds promise for combining input and output systems to construct more intricate functionalities [237]. Since the late 1960s, progress in biotechnology has bestowed the scientific community with cost-effective and temporally efficient tools for DNA extraction, sequencing, amplification, and integration of exogenous DNA elements into cells. The advent of molecular cloning and polymerase chain reaction (PCR) techniques in the 1970s and the 1980s made genetic manipulation common in microbiological research. In recognition of their pioneering work on restriction endonucleases, pivotal components of DNA synthesis, Smith, Arber, and Nathans were awarded the Nobel Prize in 1978 [238]. This recognition began a new biotechnological era, allowing the description and analysis of existing genes as well as the construction and evaluation of novel gene arrangements. Nevertheless, in the pre-genomic period, research methods categorized as genetic engineering were primarily confined to cloning and recombinant gene expression. In essence, the field of genetic engineering lacked the requisite knowledge and tools to engineer biological systems that could exhibit the diversity and intricacy of regulatory behaviors inherent in microorganisms [239, 240].

By the mid-1990s, the emergence of automated DNA sequencing and improvements in computational tools have facilitated the sequencing of entire microbial genomes [241]. As biologists and computer scientists embarked on a collaborative journey to reverse engineer cellular networks, the expanding confluence of molecular biology gave birth to the field of systems biology. In 2016, a consortium of prominent scientists proposed an ambitious synthetic biology endeavor known as the Human Genome Writing Project. This initiative leverages synthetic biology tools encompassing standardized genes, whole-genome synthesis, and CRISPR/Cas9 gene editing to craft new genomes on a substantial scale. The proposal and the emergence of systems biology have catalyzed the rapid advancement of synthetic biotechnology [242].

Synthetic biotechnology is centered on an engineering-based approach that incorporates standardized experimental methods into the iterative process of designing, modifying, and constructing synthetic biological systems to achieve predetermined goals. This facilitates the systematic engineering of biology, emphasizing standardization, quantification, and universality. This methodology transcends the inherent constraints of biological evolution, enabling the precise and purposeful synthesis of novel compounds within the natural world.

### Synthetic biology moving into biomedicine

Over the past two decades, synthetic biology has transitioned from its early role in engineering novel genetic circuits to being a pivotal component of 21st-century bioscience and biotechnology [243]. Simultaneously, the imperative to develop novel medical treatments has intensified. Synthetic biology extends the scope of traditional therapeutic interventions, offering the potential to fundamentally reshape the body’s maintenance of health and responses to diseases. CRISPR/Cas9 systems for genome engineering, gene regulatory grid analysis, and others offer a promising prospect for advanced cell-based therapies, microbiome reprogramming, and transformative disease diagnostics. The rational manipulation of bacteria through synthetic biology has given rise to the new concept of probiotics, designed to prevent and treat specific human diseases [244]. Chua et al. contended that engineered cells have the potential to address inherited or acquired metabolic disorders and target tumor cells for destruction [245]. In addition, bacterial programming may provide solutions for treating and preventing infectious diseases, offering alternatives to antibiotics and potential remedies for allergies and autoimmune disorders [240].

In summary, cells undergo modifications to produce pharmaceuticals and biofuels, the entire genome is synthesized *de novo*, and proteins and DNA molecules are endowed with non-native functionalities. Recent advances in synthetic biology have the potential to revolutionize biomedicine and biotechnology. These include the prospects of synthetic biology-based therapies to combat infectious diseases and cancer, innovations in vaccine development, microbiome manipulation, cellular therapies, and advances in regenerative medicine.

### Synthetic biology for lung disease treatment

The COVID-19 pandemic disrupted the global socioeconomic landscape, compelling biologists to seek innovative solutions [246]. Synthetic biology, with its capability to detect pathogens, administer therapeutic agents, and regulate dosages to ensure safety compliance, has facilitated diagnostic and therapeutic research in the domain of lung diseases.

#### Synthetic biology-based diagnostics

Synthetic biology techniques have been employed to innovate diagnostic technologies aimed at detecting a range of pathogens and disease biomarkers or to fabricate novel diagnostic devices [247]. Typically, synthetic biology methodologies focus on the construction of innovative biosensing systems characterized by modular architectures that encompass sensors, signal processing components, and reporting modules, all equipped with quantifiable outputs. As the field of synthetic biology evolves, most constituent elements necessary for creating biosensing systems can be readily standardized and cataloged. Significant advancements have been made in synthetic biology to manage pulmonary disorders [248]. These advances encompass various in vitro diagnostic platforms, including biosensing systems that leverage CRISPR/Cas technology and synthetic RNAs designed for the efficient identification of biomarkers linked to lung diseases [249]. Consequently, these developments hold promise for enhancing patient well-being and health outcomes.

**Fig. 7:**
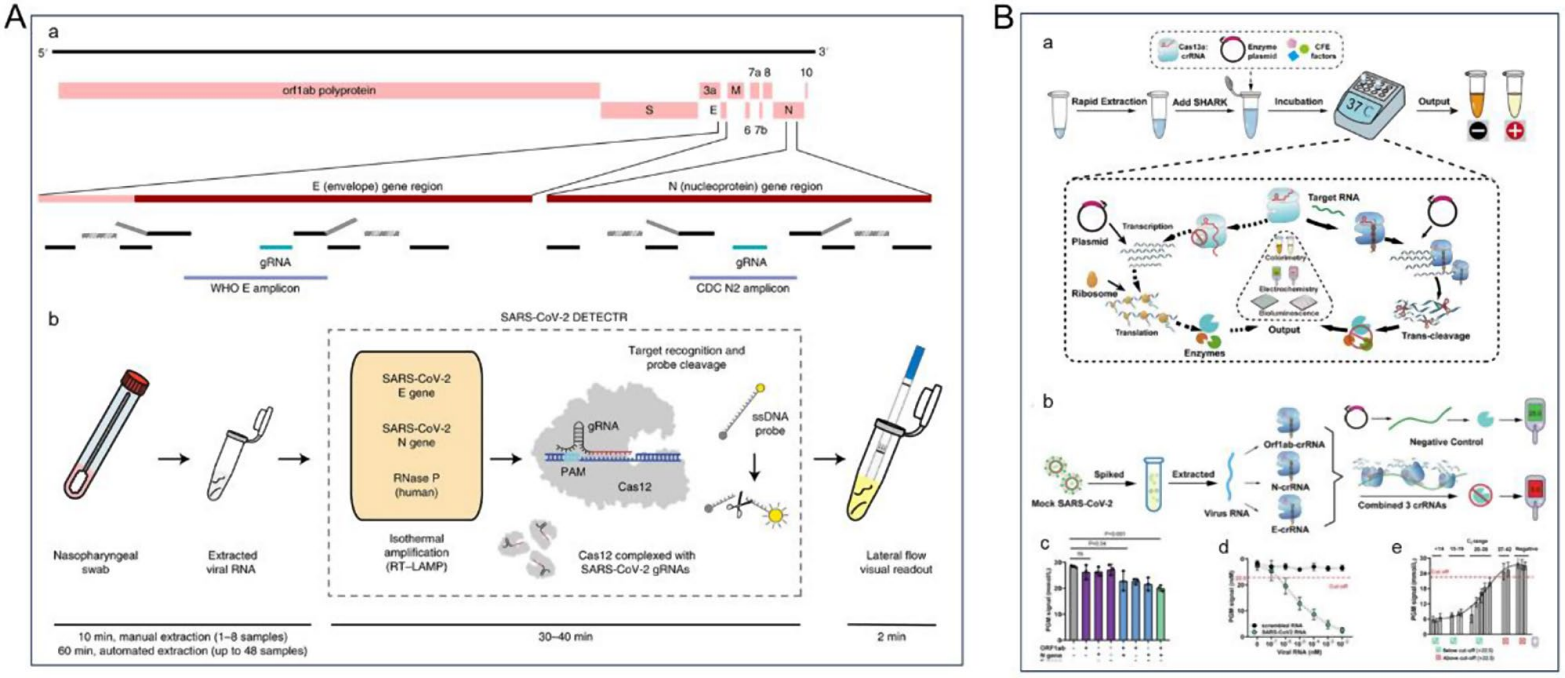
(**A**) A CRISPR/Cas12-based assay for detection of SARS-CoV-2 [250]. (a) Primers, probes, and gRNA for genome. (b) SARS-CoV-2 DETECTR workflow. (**B**) SHARK-based RNA sensing for SARS-CoV-2 detection [249]. (a) SHARK workflow. (b) SARS-CoV-2 detection. (c) Optimization of crRNA types in SHARK. (d) SHARK assay for different concentrations of viral RNAs. (e) Results based on the SHARK device assay were consistent with the Ct values of qRT-PCR. Reprinted with permission from Ref [249, 250]

CRISPR/Cas-based biosensors rely predominantly on the recognition of the disease-associated binding of specific pathogenic DNA or RNA target sequences and activate the nonspecific activity of Cas nucleases, leading to the cleavage of quenched fluorescent reporter RNAs. The cleaved RNA reporter genes subsequently emit easily detectable fluorescence signals [251]. For instance, during the identification of SARS-CoV-2 virus-specific mutations in envelope (E) and nucleoprotein (N) genes, the reporter molecule undergoes cleavage to generate a discernible viral signal [252]. To ensure reliable positive results, both genes must be detected to reduce the risk of false positives from related coronaviruses. Broughton et al. developed a CRISPR/Cas12 DETECTR technology with fast, accurate, and easy-to-use technique for the detection of SARS-CoV-2 in nasopharyngeal swab RNA extracts [250]. This method is based on the CRISPR/Cas12 lateral flow assay. The DETECTR system yielded positive results for both the E and N genes, improving the system’s accuracy in recognizing SARS-CoV-2 in the presence of other respiratory viral infections (Fig. 7A). Zhang et al. employed a cell-free, synthetic biology-driven biosensing strategy known as SHARK [249]. This approach regulates cell-free enzyme synthesis by utilizing activated Cas13a profiling to amplify RNA signals efficiently and accurately. Owing to its cascade amplification and enzyme output, SHARK offers broad compatibility in a wide range of situations. SHARK-based portable instruments have been successfully used for SARS-CoV-2 biosensing as shown in Fig. 7B, demonstrating extremely high sensitivity and selectivity, with results very close to the Ct value of quantitative reverse transcription polymerase chain reaction (qRT-PCR). Compared with existing detection methods, SHARK features precise identification, cascade amplification and customized signal output, and is promising for the development of next-generation RNA detection technologies.

The synthetic RNA biosensor module comprises an RNA switch with sequences complementary to those of the target pathogen. The binding of the target RNA initiates the expression of a reporter gene, creating visually detectable product. The method enables rapid detection and reporting of SARS-CoV-2, is simple to develop, and is cost-effective. Nuclear regulators known as programmable riboregulator toehold switches have emerged as potential detection molecules. These riboregulators are constructed from codable RNA elements that can systematically generate reporter proteins in vivo or in vitro upon interaction with target nucleic acids. This interaction assesses the status of the endogenous RNA transcripts. Koksaldi et al. successfully devised a novel riboregulatory system using in vitro synthetic biology techniques [253]. This system was used to detect the specific genomic regions of SARS-CoV-2. The presence of SARS-CoV-2-related genes triggers the translation of sfGFP mRNA, resulting in green fluorescence emission. The design of this system also facilitates the visualization of the assay results when integrated with an immediate care device. The method is direct, cost-effective, and efficient, and offers the prospect of application to SARS-CoV-2 or other viral diagnostics.

#### Synthetic biology-based therapeutics

The concept of synthetic biology entails the deliberate reconstruction and redesign of biological systems for the purpose of attaining precise objectives [18]. Its application including gene editing and gene therapy, drug development and formulation, biosensor design, and enhancement of cellular therapies. Collectively, these applications hold substantial promise for the amelioration of lung diseases, potentially ushering in a new era of treatment for pulmonary disorders while simultaneously affording innovative therapeutic modalities and approaches.

**Fig. 8:**
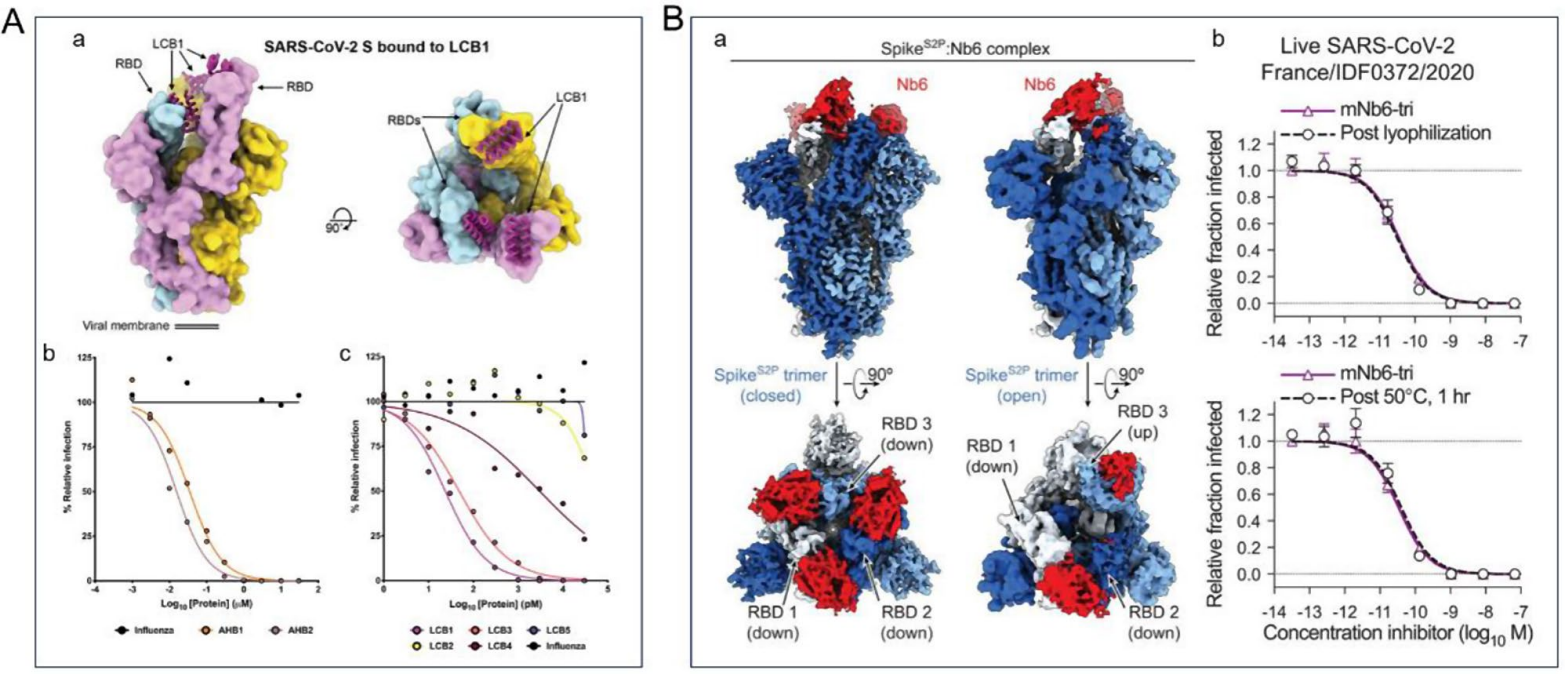
(**A**) SARS-CoV-2 miniprotein inhibitors [254]. (a) Cryo–electron microscopy structures of SARS-CoV-2 S bound to LCB1. (b-c) Design of miniprotein inhibitors to neutralize live viruses. (**B**) A trivalent nano-some that neutralizes SARS-CoV-2 by stabilizing inactivated Spike [255]. (a) Cryo–electron microscopy structures of Spike^S2P^-Nb6 complex. (b) mNb6-tri inhibits SARS-CoV-2 infection after lyophilization or heat treatment. Reprinted with permission frH

Amidst the COVID-19 pandemic, extensive analyses of vast protein sequences have been made. This endeavor seeks to identify optimal candidate proteins for synthetic vaccine development and peptidomimetic therapeutic design, with the overarching goal of advancing drug and vaccine development. The intention behind these efforts aimed to curtail the propagation of the virus and mitigate the associated morbidity and mortality attributed to COVID-19. Several compounds have been engineered, designed, and tailored for use as therapeutic agents. Cao et al. designed a high-affinity miniprotein that competes with angiotensin converting enzyme 2 (ACE2) to bind to the SARS-CoV-2 spiking receptor-binding domain [254]. The authors demonstrated the ability of miniproteins to safeguard cultured human cells from SARS-CoV-2 infection by binding to the Spike protein and efficiently neutralizing the virus (Fig. 8A). Unlike antibodies, microproteins are not expressed in the mammalian cells. Their diminutive size and heightened stability render them amenable to direct delivery into the nasal cavity or respiratory system. This investigation not only holds promise for countering the COVID-19 pandemic but also exhibits potential efficacy against a range of respiratory viral infections. In addition, Schoof et al. devised nanosomes that disrupted the interactions between Spike protein and ACE2 [255]. This was achieved by screening a library of synthetic nanosome sequences on yeast surfaces. Notably, the nanobody 6 (Nb6) interacts with Spike in an entirely inactive conformation, with its receptor-binding structural domain locked in an inaccessible downstream state, preventing it from binding ACE2 (Fig. 8B). Further refinement through affinity maturation and structure-guided design culminated in the development of a trivalent nano-some, mNb6-tri, featuring femtomolar affinity for spikes and picomolar efficacy in neutralizing SARS-CoV-2 infection. mNb6-tri maintained its functionality after nebulization, lyophilization, and heat treatment, facilitating the aerosol-mediated delivery of this potent neutralizer directly to the airway epithelium. Stability, efficacy, and ability to bind to multiple epitopes make this anti-spiking protein NP a novel approach to potentially preventing and treating COVID-19.

Although an effective antiviral drug to treat COVID-19 and other human coronavirus infections remains elusive, the FDA approved trials of Carrimycin, a synthetic biologic drug [256]. The drug is genetically engineered and modified with the incorporation of the heat-resistant Streptomyces 4-O-isopentyltransferase gene. This modification led to a notable enhancement of the antimicrobial activity of Carrimycin. In patients with severe COVID-19, Carrimycin impedes viral entry and subsequent replication events, particularly viral RNA synthesis. Studies have shown that carimycin has the highest antiviral potency and selectivity against HCoV-229E and HCoV-OC43 compared to those of acetylspiramycin and azithromycin. In addition, carimycin significantly inhibited the RNA synthesis of HCoV-OC43. However, it is not clear whether carimycin blocks viral protein synthesis, directly inhibits viral RNA synthesis, or regulates viral RNA synthesis by affecting host targets. Similar to other macrolides, carimycin can cause side effects such as adverse gastrointestinal reactions, but most are mildly tolerable.

Given the lack of potent antiviral drugs and effective therapeutic interventions for COVID-19, vaccination has emerged as the most effective control measure. Various strategies have been employed to develop SARS-CoV-2 vaccines such as DNA- and RNA-based [257]. Synthetic mRNA-based vaccines are particularly promising, being the first FDA-approved COVID-19 vaccine [258]. mRNA vaccines are lipid-based nanoparticle nucleotide-modified vaccines that employ viral proteins to induce immune responses during antigen presentation. SARS-CoV-2 involves viral spike proteins that bind to host cell ACE2 receptors and subsequently generate neutralizing antibodies in the presence of the virus [259]. Unlike traditional approaches, mRNA production facilitates large-scale vaccine manufacturing, thereby addressing the need for mass vaccination. This approach offers multiple advantages, including safety, cost-effectiveness, and the elicitation of both cell- and antibody-mediated immune responses.

In conclusion, since the 2019 epidemic, synthetic biotechnology has excelled in the development of diagnostics, therapeutics, and vaccines. However, it still has practical problems and technical challenges. For example, issues such as how to effectively avoid off-target effects, improve editing efficiency, and reduce immunogenicity limit the broad application of synthetic biotechnology in clinical diagnosis and treatment. In addition, targeted synthetic biotechnology can be used to design microorganisms or viruses with new functions. While these new organisms may have potential applications, they may also be misused or released into the environment with unpredictable consequences. Therefore, strict safety standards and regulatory mechanisms are therefore needed to ensure that the development of synthetic biology does not pose potential safety risks. In addition, synthetic biology designs and modifies organisms on a large scale, which may raise several ethical issues, such as the moral and social implications of human gene editing and the definition and boundaries of synthetic life. Hence, future research and applications of synthetic biology should pay attention to ethical considerations and sustainability to ensure that their development of synthetic biology technology is consistent with social values and environmental protection.

## Summary and outlook

This review explored the use of nanomaterial-assisted gene editing and synthetic biology in diagnosing and treating lung diseases. The intricacies of the respiratory system often pose challenges to the management of lung diseases. Advances in nanotechnology have led to nanocarrier-based nano-delivery systems that can enhance lung biocarrier penetration through strategic considerations such as surface properties, particle size and shape. Consequently, the combination of nanotechnology and pulmonary drug delivery is a promising strategy for enhancing drug efficacy, release, and therapeutic effectiveness.

To enhance the efficacy of lung-targeted therapies, researchers have been actively exploring combining the unique properties of nanomaterials with gene editing techniques to achieve more precise and effective gene therapies. Among the gene editing systems, the emergence of the CRISPR/Cas9 system has greatly simplified the gene editing procedure and has had a profound impact on molecular biology and gene therapy. These proteins also function as nuclease enzymes to edit the genome of the target cell. Traditionally, delivery of the CRISPR/Cas9 gene-editing system relied on viral vectors. However, the immunogenicity, cytotoxicity, and potential infection risks associated with viral vectors limit their suitability as gene delivery vectors. Significantly, the rise of surface-modified or functionalized materials has propelled nanomaterials to the forefront as preferred carrier materials for non-viral CRISPR/Cas9 delivery systems. This is attributed to their high gene-editing efficiency, tissue/cell specificity, and low immunogenicity.

This review provides a comprehensive survey of nanomaterials as carriers for gene editing system delivery and their applications in lung disease therapy. The successful application of diverse biomaterials in CRISPR/Cas9 delivery systems underscores their versatility and adjustability, rendering them attractive solutions for addressing the various biological challenges associated with CRISPR/Cas9 delivery. Despite the advantages of biomaterial carriers, challenges remain in the delivery of CRISPR/Cas9 systems. Issues include achieving NP enrichment at precise locations, shielding the CRISPR/Cas9 system from detection and clearance by the reticuloendothelial system, and facilitating carrier penetration of a hydrophobic cell membrane. Furthermore, the immunological complications linked to biomaterial CRISPR/Cas9 systems pose significant concerns. With the advent of new biomaterial technologies and the intensive study of multiple extracellular and intracellular delivery systems, these limitations are expected to be progressively addressed by interdisciplinary researchers. The increasing efficiency of biomaterial delivery will bolster the clinical translation of CRISPR/Cas9 technology.

In the wake of the COVID-19 pandemic, it has become evident that traditional methods for detecting and diagnosing infectious pathogens have various limitations. These include dependence on well-established and comprehensive laboratories, the necessity for qualified personnel, the absence of standardized protocols, time-consuming processes, and increased susceptibility to false-negative and false-positive results. Consequently, these conventional analytical methods cannot provide reliable point-of-care testing solutions. Synthetic biology has emerged as a formidable asset in the medical field, in both industrialized and low-income countries. It excels in enhancing technology and point-of-care testing and addresses the drawbacks of traditional diagnostic approaches in the battle against deadly disease outbreaks. As an emerging interdisciplinary discipline, synthetic biology aims to realize the precision and modification of biological systems through in-depth research and engineering of organisms. This interdisciplinary approach enables the programming of microorganisms to provide swift, precise, specific, cost-effective, and non-invasive modalities in diagnosing and treating infectious diseases.

In the realms of nanomaterials, gene editing, and synthetic biology, advancements within a discipline have yielded novel tools and insights. The convergence of these two fields constitutes a prominent trend in their development, owing to the distinctive attributes and requirements of synthetic biology and nanobiology. With the continuous development of nanotechnology, arrays of nanoscale components can serve as catalysts, sensors, and delivery vehicles. This enables the intricate construction of gene circuits, precise regulation of gene circuit operation, and the execution of in vivo gene editing and modification. Synthetic biology can orchestrate specific biomolecules and ecosystems according to human design, autonomously produce nanomaterials, and yield nano-preparations, while simultaneously enhancing their efficiency and reducing toxicity.

Consequently, the interdisciplinary intersection and integration of nanotechnology, gene editing, and synthetic biology have emerged as significant focal points for the growth of these disciplines. Future advances in this convergence should focus primarily on achieving standardization and modularization. Standardization forms the cornerstone of the efficient construction and operation of nanodevices, while modularity of biological components is a fundamental feature of synthetic biology. Designing standardized, universally applicable interfaces and regulatory elements enables the targeted integration of functional modules, facilitating the scalability of synthetic biological systems. Moreover, the development of analytical techniques is imperative, encompassing multidisciplinary approaches, such as multimodal imaging techniques, fluorescence resonance energy transfer, and multi-omics analysis. These techniques are urgently needed for the in vivo monitoring and assessment of nanosynthesized biological systems. Furthermore, investigations into the biodistribution, effects on nontargeted tissues, and metabolic fate of synthetic nanobiosystems are essential to lay the groundwork for clinical translation and large-scale production applications.

## Data Availability

No datasets were generated or analysed during the current study.
